# Multigene Germline Panel Testing in Gastric Cancer Patients in a Portuguese Population

**DOI:** 10.1002/cam4.71732

**Published:** 2026-03-19

**Authors:** B. Mourato, B. Cordeiro, F. Costa Pinto, N. Pratas, H. Capote, I. Fronteira, M. Areia, R. Dinis

**Affiliations:** ^1^ NOVA National School of Public Health, Public Health Research Centre, Comprehensive Health Research Center, CHRC, LA‐REAL, CCAL, NOVA University Lisbon, Lisbon, Portugal Lisbon Portugal; ^2^ Unidade Local de Saúde Do Alto Alentejo, Hospital Doutor José Maria Grande Portalegre Portugal; ^3^ Instituto Português De Oncologia De Coimbra Coimbra Portugal; ^4^ RISE@CI‐IPO (Health Research Network) Portuguese Oncology Institute of Porto (IPO Porto) Porto Portugal; ^5^ Hospital Do Espírito Santo De Évora Évora Portugal

**Keywords:** early detection of cancer, gastrointestinal neoplasms, germ‐line mutation, hereditary, neoplastic syndromes, stomach neoplasms

## Abstract

**Background:**

Gastric cancer is a heterogeneous disease with both environmental and genetic determinants. While Multigene Germline Panel Testing (MGPT) increasingly reveals hereditary predisposition, the prevalence and clinical significance of germline variants in Portuguese gastric cancer patients tested through institutional MGPT programs remain insufficiently characterised. The main objective of this study was to determine the prevalence and spectrum of germline pathogenic variants and likely pathogenic variants in gastric cancer patients from Portugal, and to explore their clinicopathological correlations.

**Methods:**

We conducted a pilot retrospective observational study including 51 patients with histologically confirmed gastric cancer who underwent MGPT between 2020 and 2025. Genetic testing was performed using validated 15 or 30 gene panels. Clinical, pathological, and survival data were retrieved from medical records.

**Results:**

Germline pathogenic or likely pathogenic variants were detected in 6 patients (11.8%), involving MLH1, MSH6, PMS2, CHEK2, and BLM. Variants of uncertain significance (VUS) were identified in 11.8%. No CDH1 alterations were observed. Compared with MGPT‐negative cases, MGPT‐positive patients were significantly younger at diagnosis (median 48 vs. 76 years, *p* = 0.00) and more frequently presented with mixed‐type gastric cancer (33.3% vs. 4.4%, *p* = 0.05). No significant associations were found with tumour stage or treatment.

**Conclusions:**

In this Portuguese cohort, nearly 12% of consecutive real‐world MGPT‐tested gastric cancer patients had germline pathogenic or likely pathogenic variants, predominantly in mismatch repair genes but also in CHEK2 and BLM. These findings further illustrate the heterogeneity of hereditary gastric cancer beyond classical CDH1 mutations and support the integration of MGPT into clinical care to identify at‐risk individuals not captured by current criteria. Larger multicentre studies are warranted to validate these results and clarify the role of emerging susceptibility genes.

**Trial Registration:**

Study Registration Number: NCT07387237

**Abbreviations**
HDGC: Hereditary Diffuse Gastric Cancer
MGPT: Multigene Germline Panel Test
VUS: Variants of Uncertain Significance

## 1 Introduction

In 2022, data from the Global Cancer Observatory revealed that gastric cancer was the fifth cancer regarding incidence and mortality worldwide [1].

It is a multifactorial and heterogeneous disease, where both environmental and genetic factors are involved. Although the majority of cases are sporadic, caused by environmental exposure, up to 20% of patients have a familial aggregation and of these, 3 to 5% are classified as hereditary, with an identified mutation [2, 3].

Three hereditary syndromes mainly affect the stomach: Hereditary Diffuse Gastric Cancer (HDGC); Gastric Adenocarcinoma and Stomach Proximal Polyposis; and Familial Intestinal Gastric Cancer [4]. HDGC is an autosomal dominant syndrome, predominantly caused by germline mutations in the CDH1 gene, which encodes the e‐cadherin protein with important functions in cell aggregation, characterised by a high‐risk of early‐onset diffuse gastric cancer and invasive lobular breast cancer [5]. Gastric Adenocarcinoma and Stomach Proximal Polyposis is caused by germline mutations in APC promotor 1B and is characterised by the existence of fundic gland polyposis with focal dysplasia and intestinal or mixed adenocarcinoma at the level of the gastric fundus and sparing the antrum [6, 7]. Finally, Familial Intestinal Gastric Cancer is an autosomal dominant syndrome, still poorly genetically characterised, which is associated with an increased risk of intestinal gastric cancer [5]. In addition, other genetic syndromes have an increased risk of gastric cancer. Some of the genes involved include MLH1, MSH2, MSH6, and PMS2 (Lynch Syndrome), TP53 (Li‐Fraumeni Syndrome), APC (Familial Adenomatous Polyposis and proximal polyposis of the stomach) and MUTYH (Polyposis Associated MUTYH), BMPR1A and SMAD4 (Juvenile Polyposis Syndrome), STK11 (Peutz‐Jeghers Syndrome) and PTEN (Cowden Syndrome) [6, 8, 9]. As gastric cancer is a neoplasm with numerous somatic and hereditary mutations involved in its carcinogenesis, the use of Multigene Germline Panel Testing (MGPT) can be decisive, as it performs the analysis of hereditary mutations in a significant number of genes simultaneously (depending on the genes that integrate the panel) [10, 11]. In addition to the described mutations, MGPT has allowed the identification of new genetic mutations with risk for gastric cancer, including in the BRCA 1/2, PALB2, ATM and RAD51C genes (typically associated with hereditary breast and ovarian cancer), MAP3K6, MYD88, among others [3, 12, 13, 14, 15, 16, 17]. However, the use of MGPT, especially in *broader gastric cancer populations*, is not without risks, an example being the unexpected detection of pathogenic or likely pathogenic variants in genes not strictly associated with the phenotype (secondary findings) or, more frequently, the detection of variants of uncertain significance (VUS), which can raise important questions regarding the uncertainty of how to approach these patients [18]. Therefore, several studies have emerged that only offer MGPT or the analysis of a reduced number of genes (single gene test) to selected patients, particularly with suspected HDCG [9, 19, 20, 21]. On the other hand, the evidence of many other germline mutations associated with gastric cancer and the lack of genetic characterisation of Familial Intestinal Gastric Cancer justifies the growing interest in performing MGPT beyond non‐phenotype‐restricted gastric cancer populations [5, 18, 22, 23]. Although these studies point to a benefit in the use of MGPT in *non–criteria‐restricted gastric cancer populations*, this test is not yet conducted in a widespread manner.

In Portugal, gastric cancer incidence and mortality rates have been increasing over the last few decades [1]. Therefore, it is important to better understand the genetic characteristics of gastric cancer in this country, in order to develop better strategies to approach this disease. The main objective of this research was to quantify and characterise the pathogenic variants found in MGPT of consecutive real‐world gastric cancer patients in a specific Portuguese population (Alto Alentejo), and explore their clinicopathological correlations.

## 2 Materials and Methods

We conducted a pilot retrospective observational study at the Unidade Local de Saúde do Alto Alentejo, Portugal. All MGPT was performed in the same accredited laboratory (Germano de Sousa—Centro de Genética Laboratorial) using peripheral blood samples (Data S1).

Eligible participants were adults (≥ 18 years) with histologically confirmed gastric adenocarcinoma who underwent MGPT between 2020 and 2025. Additional requirements included ability to understand Portuguese and/or English and to provide valid informed consent. Patients without a definitive genetic report or with incomplete clinical data for key study variables were excluded. All patients who met eligibility and inclusion criteria were included in the study.

During the study period, MGPT was systematically proposed to all newly diagnosed gastric cancer patients treated at our institution as part of a pilot clinical initiative. In accordance with institutional administrative procedures within the public healthcare system, all genetic testing requests were formally discussed at multidisciplinary tumour board meetings to allow documentation and approval of testing, rather than to select patients for eligibility. Consequently, the study cohort represents consecutive gastric cancer patients undergoing germline testing within routine institutional practice during the implementation phase of MGPT.

Two different MGPT panels were applied: a 15‐gene panel (code 4005) and a 30‐gene panel (code 4013) (Data S1), with the choice of panel determined during discussion at the multidisciplinary tumour board. The choice of panel followed institutional clinical practice, whereby patients presenting additional personal or familial oncological history suggestive of hereditary cancer predisposition were preferentially offered the expanded 30‐gene panel. MGPT was offered as part of institutional routine practice and was not restricted to patients meeting predefined hereditary gastric cancer clinical criteria.

MGPT was considered positive when at least one pathogenic or likely pathogenic germline variant was identified in any gene included in the panel. Variants of uncertain significance (VUS) were not regarded as positive for the primary analysis.

Clinical information was collected through structured interviews and complemented by review of electronic health records.

### 2.1 Statistical Analysis

All statistical analyses were conducted using SPSS (version 29.0.2.0). Results are reported by mean and standard deviation for normally distributed continuous variables; median and interquartile range for non‐normal distributions; absolute and relative frequencies for categorical variables. For bivariate comparisons Student's *t*‐test was applied for normally distributed continuous variables, and the Mann–Whitney U test for non‐normal distributions. Associations between categorical variables were evaluated with Pearson's chi‐square or Fisher's exact test, as appropriate.

### 2.2 Ethical Considerations

The study was conducted in accordance with the Declaration of Helsinki and was approved by the Ethics Committee of the Institution (Data S2 and S3). All data were Anonymised and managed in compliance with the General Data Protection Regulation.

## 3 Results

### 3.1 Patient Characteristics

A total of 51 patients with histologically confirmed gastric adenocarcinoma were included (Data S4). The median age at diagnosis was 75 years (Data S5 and S6 Age statistics/BMI Statistics) and the majority of patients were male (70.6%) (Table 1).

**TABLE 1:** Population demographics (IQR: Interquartile range, SD: Standard deviation).

Characteristic	Value
Age at diagnosis, years	Median 75.0 (IQR 67–81; range 32–91)
Sex	Male 36 (70.6%)
Body mass index, kg/m^2^	Mean 24.7 (SD 4.5; range 12.5–33.0)
Previous oncological disease	11 (21.6%)
Comorbidities	46 (90.2%)
Cardiovascular	36 (70.6%)
Psychiatric/Neurologic	13 (25.5%)
Gastrointestinal	11 (21.6%)
Osteoarticular/Rheumatologic	10 (19.6%)
Reproductive System	7 (13.7%)
Pulmonary	5 (9.8%)
Alcohol/tobacco dependence	3 (5.9%)
Family history of cancer	31 (60.8%)
Family history of gastric cancer	11 (35.5%)
More than 1 relative with cancer	18 (58.1%)

Comorbidities were frequent (90.2%), particularly cardiovascular (70.6%), psychiatric/neurological (25.5%) and gastrointestinal disorders (21.6%) (Table 1).

A previous history of another malignancy was reported in 21.6%, whereas in 78.4% the gastric cancer as their only oncological diagnosis. A positive family history of cancer was present in 60.8%, of whom 35.5% were diagnosed with gastric cancer (Table 1); when stratifying these familial gastric cancer cases by histologic subtype, 81.8% were of the intestinal type (9 out of 11), with one diffuse and one mixed tumour. No significant association was found between familial aggregation of gastric cancer and histologic subtype (*p* = 0.61) (Data S7—Histological subtype in familial gastric cancer). Moreover, 18 patients (58.1%) reported more than one relative affected by any type of cancer.

### 3.2 Gastric Cancer Characteristics

Among 51 patients, tumours were most often located in the antrum (25.5%) or body (23.5%) and were predominantly intestinal‐type (72.5%); clinical stage distribution (National Comprehensive Cancer Network criteria) ranged I–IV (31.4%, 25.4%, 21.6% and 17.6%, respectively), 80.4% underwent oncological resection (mostly subtotal gastrectomy), 51.0% received peri‐operative chemotherapy and 19.6% palliative chemotherapy. Table 2 presents the summary of gastric cancer characteristics in our cohort.

**TABLE 2:** Gastric Cancer characteristics.

Characteristic	*n* (%)
Tumour location	
Antrum	13 (25.5)
Body	12 (23.5)
Body + antrum	9 (17.6)
Incisura	5 (9.8)
Pylorus	4 (7.8)
Cardia	3 (5.9)
Fundus	1 (2.0)
Cardia + fundus	1 (2.0)
Cardia + body + antrum	1 (2.0)
Antrum + pylorus	1 (2.0)
Unknown	1 (2.0)
Histology (Lauren classification)	
Intestinal	37 (72.5)
Diffuse	10 (19.6)
Mixed	4 (7.8)
Clinical stage	
I	16 (31.4)
IIA	9 (17.6)
IIB	4 (7.8)
III	11 (21.6)
IVB	9 (17.6)
Surgical treatment	41 (80.4)
Subtotal gastrectomy	36 (87.8)
Total gastrectomy	5 (12.2)
Pathological stage	
0	4 (7.8)
IA	7 (13.7)
IB	9 (17.6)
IIA	9 (17.6)
IIB	6 (11.8)
IIIA	2 (3.9)
IIIC	1 (2.0)
IV	2 (3.9)
Chemotherapy	
Peri‐operative	26 (51.0)
Palliative	10 (19.6)
None	15 (29.4)

At 12 month follow‐up, 74.5% patients (*n* = 38) were alive, 9.8% had died (*n* = 5), and 15.7% (*n* = 8) had not yet reached 1 year after diagnosis. The mean overall survival time since diagnosis was 2.16 years (SD 1.7; range 0.2–5 years).

### 3.3 MGPT Results

Two different MGPT panels were applied: A 15‐gene panel (code 4005) in 36 patients (70.6%) and a 30‐gene panel (code 4013) in 15 patients (29.4%).

Overall, pathogenic variants were detected in 4 patients (7.8%), and likely pathogenic variants in 2 patients (3.9%), accounting for a total of 6 patients (11.8%) harbouring germline relevant alterations. In addition, variants of uncertain significance (VUS) were identified in 6 patients (11.8%).

**FIGURE 1:**
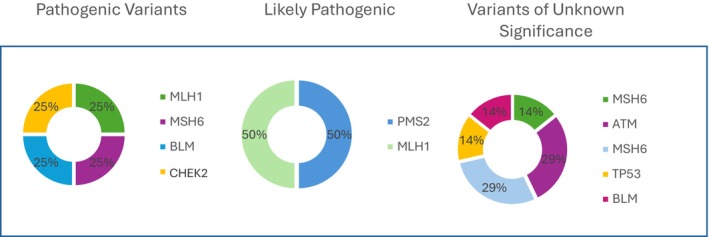
Spectrum of identified genetic variants.

Pathogenic variants were found in MLH1, MSH6, CHEK2, and BLM, whereas likely pathogenic variants were identified in PMS2 and MLH1 (Figure 1). The specific variants detected included MLH1 (c.1409 + 2 T > G), MSH6 (c.3261del), CHEK2 (c.592 + 3A > T), MLH1 p.Ala681Thr (c.2041G > A), BLM (c.2206dup), and PMS2 c.1204C > T; p (Gln402Ter). In addition, variants of uncertain significance were detected in 6 cases, involving BLM, MSH6, ATM, and TP53.

Table 3 resumes all cases with positive variants, pathogenic, likely pathogenic, or of unknown significance, and respective main clinical features.

**TABLE 3:** Cases with pathogenic variants (PV), likely pathogenic (LP) or variants of unknown significance (VUS). cStage: Clinical stage: PStage: Pathological stage.

Age	Sex	Histology	Stage	Gene/variant	Variant type
44	M	Intestinal	pStage: IIA	MLH1 (c1409 + 2 T > G)	PV
77	M	Intestinal	pStage: IIA	MSH6 (c.3261del.p)	PV
44	M	Mixed	pStage: IIIA	CHEK2 (c.592 + 3A > T)	PV
66	F	Diffuse	pStage: IIB	BLM c.2206dup p (Tyr736LeufsTer5); c.2208_2212delTCTGAinsGATTC p (Tyr736Ter)	PV
52	M	Intestinal		PMS2 (c.1204C > T p (Gln402Ter))	LP
32	M	Mixed	pStage: IV	MLH1 p.Ala681Thr (c.2041G > A)	LP
55	M	Diffuse	pStage: IIB	BLM c.842A > C p (His281Pro)	VUS
52	M	Intestinal	cStage	MSH6 (c.3452C > G p (Ala1151Gly))	VUS
88	F	Intestinal	pStage: IB	ATM (c.4414 T > G p (Leu1472Val))	VUS
79	M	Intestinal	pStage: IB	MSH6 (c.3542A > G p (Asp1181Gly))	VUS
68	M	Diffuse	pStage: IB	ATM c.5821G > C p (Val1941Leu); TP53 c.1177G > T p (Asp393Tyr); BLM c.2720C > T p (Thr907Met)	VUS
55	F	Diffuse	pStage: IIB	MSH6 c.3134A > G p (Lys1045Arg)	VUS

At the variant level, MLH1 (c.1409 + 2 T > G) represented a canonical splice‐site mutation predicted to disrupt mRNA splicing and abolish protein function; MSH6 (c.3261del) was a frameshift deletion introducing a premature stop codon; and PMS2 (c.1204C > T; p (Gln402Ter)) a nonsense mutation, all consistent with Lynch syndrome–associated pathogenicity. In addition, CHEK2 (c.592 + 3A > T), a splice‐site variant, may impair DNA damage response signalling, while BLM (c.2206dup), a frameshift duplication, is predicted to impair helicase activity and genomic stability.

### 3.4 Correlation Between MGPT Results and Population Characteristics

The analysis of demographic variables revealed that MGPT‐positive patients were diagnosed at a significantly younger age compared with MGPT‐negative individuals (median age 48 vs. 76 years, *p* = 0.00) (Data S8—MGPT with Age). With regard to sex distribution, MGPT‐positive cases were predominantly male but without statistical significance (83.3% vs. 68.9%, *p* = 0.42) (Data S9 MGPT with Sex). The mean BMI did not differ significantly between MGPT‐positive and MGPT‐negative patients (25.5 vs. 24.6 kg/m^2^, *p* = 0.38) (Data S10 MGPT with BMI).

No significant associations were observed between MGPT‐positive versus negative patients: cardiovascular (73% vs. 50%, *p* = 0.23), psychiatric/neurologic (33% vs. 24%, *p* = 0.48), gastrointestinal (17% vs. 22%, *p* = 0.62), osteoarticular/rheumatologic (0% vs. 22%, *p* = 0.25), reproductive system (0% vs. 16%, *p* = 0.39), pulmonary (0% vs. 11%, *p* = 0.52), and alcohol/tobacco‐related disorders (0% vs. 7%, *p* = 0.68) (Data S11 MGPT with Comorbidities).

A previous history of malignancy was reported in half of MGPT‐positive patients compared with 17.8% of MGPT‐negative individuals. Although this result suggests a potential enrichment of germline variants among patients with multiple primary cancers, the difference did not reach statistical significance (*p* = 0.07) (Data S12 MGPT with other cancers in same patient).

No significant associations were identified between MGPT‐positive versus negative patients and family history of cancer. A direct relative with gastric cancer was reported in 66.7% vs. 60.0% (*p* = 0.75) (Data S13 MGPT with familial cancers); similarly, having more than one relative affected by cancer (16.7% vs. 37.8%, *p* = 0.31) or meeting the criteria for familial gastric cancer (16.7% vs. 22.2%, *p* = 0.76) showed no meaningful differences (Data S13 MGPT with familial cancers).

### 3.5 Correlations Between MGPT Results and Tumour Characteristics, Treatment, and Prognostic Factors

When analysing tumour‐related features, the distribution of histological subtypes differed significantly between MGPT‐positive and MGPT‐negative patients (*p* = 0.046). Among MGPT‐positive cases, intestinal‐type gastric cancer was less frequent (50.0% vs 75.6%), while mixed‐type tumours were considerably more common (33.3% vs 4.4%). The proportion of diffuse‐type cancers was similar between groups (16.7% vs 20.0%) (Data S14—MGPT with histological subtypes). Comparing MGPT‐positive versus negative patients, different distributions across gastric subsites (*p* = 0.019). Antral tumours were similarly frequent in both groups (50.0% vs 46.7%), while non‐antral locations predominated among MGPT‐negative cases (53.3% vs 33.3%) (Data S15—MGPT Antrum vs Non Antrum). No significant differences were observed between the two groups regarding clinical stage (stage IV vs. stages I‐III 33.3% vs. 15.6%, *p* = 0.07) (Data S16—MGPT and Stage IV vs I‐III), surgical management (total gastrectomy 16.7% vs. 8.9%, *p* = 0.29) (Data S17—MGPT and type of surgery), need for chemotherapy (66.7% vs. 71.1%, *p* = 0.58) (Data S18—MGPT and need of QT). Twelve‐month survival did not differ significantly between MGPT‐positive and MGPT‐negative patients (66.7% vs 75.6%, *p* = 0.08) (Data S19—MGPT with Prognosis).

Among the six patients with pathogenic or likely pathogenic variants, none fulfilled Amsterdam II or revised Bethesda criteria for Lynch syndrome. Detailed clinical and family history characteristics are provided in Data S20—Amsterdam II and revised Bethesda criteria for Lynch syndrome in MGPT‐positives.

## 4 Discussion

This study should be interpreted as a pilot real‐world cohort reflecting early institutional implementation of MGPT in gastric cancer patients.

Germline pathogenic or likely pathogenic variants were identified in 11.8% of consecutive real‐world MGPT‐tested Portuguese gastric cancer patients, predominantly affecting mismatch repair genes (MLH1, MSH6, PMS2) together with variants in CHEK2 and BLM. No CDH1 alterations were detected, consistent with evidence that this gene is rarely involved outside families meeting criteria for HDGC. These findings reinforce the heterogeneity of hereditary gastric cancer beyond classical HDGC genes.

Together, these variant‐level observations reinforce a predominant role for mismatch‐repair deficiency in hereditary gastric cancer susceptibility—particularly MLH1, which demonstrated the most consistent association with intestinal‐type histology—whereas MSH6 and PMS2 exhibited lower gastric penetrance. By contrast, CHEK2 and BLM were identified in single carriers and their contribution to gastric carcinogenesis remains uncertain. Clinically, pathogenic mismatch‐repair variants should prompt referral for genetic counselling and implementation of established colorectal surveillance, with consideration of targeted gastric surveillance in families or populations at increased gastric cancer incidence; specific surveillance pathways for CHEK2/BLM carriers are not supported by current evidence. These findings are limited by small carrier numbers, heterogeneity of panel composition, retrospective design and lack of functional validation for several variants, and therefore require confirmation in larger, prospectively ascertained cohorts.

Comparable prevalence has been reported internationally: 13.5% in the Italian experience of Calvello et al. [18], 15.6% in the multicentre Uson et al. [22] study, and 6.2% in the Kyrgyz cohort of Bilyalov et al. [23]. More recently, Gilad et al. [24] reported 13.3% across 3700 gastric cancer patients, with a predominance of homologous recombination and hereditary diffuse gastric associated genes, while a Chinese multicentre study of high‐risk cases observed 25% germline carriers across a broader panel [25]. Collectively, these studies confirm that prevalence is relatively consistent worldwide (6%–25%) but that the gene spectrum varies by geography, testing strategy, and patient selection.

A recent large multicentre study from Latin America and Europe reported a 6% prevalence of pathogenic/likely pathogenic variants in multi‐organ cancer predisposition genes [26]. Notably, many carriers did not fulfil established genetic testing criteria. Our findings are consistent with these observations and further contribute population‐specific data from an intermediate‐risk Portuguese setting. Unlike that cohort, which included early‐onset and/or family‐history enriched cases, our study reflects consecutive real‐world MGPT testing in a regional institution, thereby providing complementary evidence regarding the potential value of broader germline testing strategies.

The absence of homologous recombination repair genes such as BRCA1, BRCA2, and PALB2 in the initial 15‐gene panel may have resulted in underestimation of the true prevalence of pathogenic variants. Therefore, our reported prevalence should be interpreted as conservative.

Clinical and pathological correlations in our series revealed distinct features among MGPT‐positive patients. Most notably, carriers of pathogenic or likely pathogenic variants were diagnosed at a significantly younger age compared with MGPT‐negative individuals (median 48 vs. 76 years, *p* = 0.00), supporting the concept that hereditary predisposition contributes to early‐onset gastric cancer [3]. In addition, MGPT‐positive tumours more frequently exhibited a mixed histologic subtype (33.3% vs. 4.4%, *p* = 0.05), while MGPT‐negative tumours are more frequently intestinal adenocarcinoma (75.6% vs 50%, *p* = 0.05). This observation, although based on small numbers, may warrant further exploration in larger cohorts, since mixed and diffuse gastric adenocarcinomas are typically more aggressive than intestinal types, characterised by infiltrative growth, early peritoneal dissemination, and poorer prognosis [8].

No significant differences were observed regarding clinical or pathological stage, or receipt of chemotherapy, reflecting the heterogeneity of gastric cancer presentation across hereditary and sporadic settings. No statistically robust survival differences were identified. Given the small number of variant carriers and limited follow‐up, prognostic inference is not appropriate.

Our findings have several important clinical implications. First, they underscore the value of MGPT in consecutive real‐world gastric cancer patients, as a relevant proportion of pathogenic variants would have been missed by applying conventional clinical criteria alone [22]. This observation is consistent with recent international data and suggests that routine incorporation of MGPT could expand the identification of at‐risk individuals and enable targeted surveillance strategies for their relatives. In particular, the detection of mismatch repair gene variants has direct implications for Lynch syndrome diagnosis, surveillance of the upper gastrointestinal tract, and cascade testing within affected families. Notably, none of the MMR variant carriers fulfilled classical Amsterdam II or revised Bethesda criteria, underscoring the potential value of broader germline testing strategies beyond traditional clinical selection tools.

The identification of variants in CHEK2 and BLM also raises relevant questions. Although their role in gastric cancer predisposition remains less well established, accumulating evidence points toward a broader panel of genes contributing to hereditary susceptibility [24, 25]. This highlights the need for ongoing data collection and collaborative studies to refine the clinical management of patients carrying such variants.

In our cohort, VUS were detected in 11.8% of patients, involving DNA repair–related genes. Although lacking current clinical utility, these findings highlight the interpretative challenges inherent to MGPT. Reported rates in the literature vary widely, ranging from 10% to over 30%, depending on the breadth of panels applied and population heterogeneity [18, 22, 23]. VUS require careful communication during genetic counselling and periodic reassessment as classification evolves. Collaborative databases are essential to improve variant interpretation and to clarify the potential contribution of emerging genes to gastric cancer susceptibility.

Nevertheless, our study has several limitations. The retrospective design and relatively small sample size limit statistical power and the ability to detect subtle associations. Being a single‐centre cohort from a specific Portuguese region, our findings may not be generalizable to the broader national or international populations. However, as all consecutive patients with histologically confirmed gastric adenocarcinoma who met inclusion criteria were offered germline multigene panel testing as part of institutional practice, the cohort reflects the total eligible gastric adenocarcinoma cases within the region during the study period, minimising selection bias at the institutional level.

The use of different panel sizes (15 vs. 30 genes) introduces heterogeneity in variant detection and may have influenced the overall detection rate. Importantly, all patients underwent at least the same 15‐gene core panel encompassing the principal high‐penetrance gastric cancer susceptibility genes, providing a consistent baseline genetic assessment across the cohort.

To further explore the potential influence of panel heterogeneity, an exploratory descriptive comparison of pathogenic variant detection according to panel size was performed Data S21. Detection of pathogenic or likely pathogenic variants according to MGPT panel size. Although detection rates were higher among patients tested with the expanded panel, these findings should be interpreted cautiously given the clinically driven allocation of expanded testing.

Survival analyses were limited by short follow‐up and small numbers of events, restricting prognostic inference. Selection bias may also be present, as only patients with available MGPT reports were included, and incomplete clinical data could not always be excluded.

Given the limited number of variant‐positive patients, the study is underpowered to detect prognostic associations, and the risk of Type II error should be acknowledged.

Correlation between germline mismatch repair variants and tumour MMR deficiency assessed by microsatellite instability or immunohistochemistry was not systematically available in this retrospective cohort. Integration of germline and somatic molecular data represents an important future research direction to further clarify the biological and clinical significance of hereditary findings in gastric cancer.

Finally, the lack of an external comparator cohort hinders contextualization of our prevalence estimates within the broader Portuguese or European setting. Future studies should aim to validate these observations in larger, multicentre Portuguese and/or International cohorts, with standardised panel sizes, prospective design, longer follow‐up, and integration of genetic counselling, to inform the role of MGPT in clinical practice guidelines. Therefore, the present findings should be interpreted as hypothesis‐generating rather than confirmatory.

## 5 Conclusions

In this Portuguese cohort of consecutive real‐world MGPT‐tested gastric cancer patients, germline pathogenic or likely pathogenic variants were identified in 11.8% of cases, most frequently in mismatch repair genes, but also in CHEK2 and BLM. These findings further support a meaningful contribution of hereditary susceptibility to gastric cancer beyond classical HDGC and highlight the genetic heterogeneity of this disease.

Our results support continued evaluation of MGPT as a tool to identify at‐risk individuals who would not be captured by current clinical criteria, with eventual implications for patient management, family counselling, and surveillance strategies. Larger multicentre studies are needed to validate these observations, clarify the role of emerging susceptibility genes, and guide the integration of germline testing into gastric cancer care pathways.

## Author Contributions

**B. Mourato:** conceptualization, investigation, writing – original draft, methodology, formal analysis, data curation. **M. Areia:** conceptualization, writing – review and editing, supervision.

## Funding

The costs associated with the submission and potential publication of this manuscript will be covered by the lead author and her research consultancy, TrueConnection Lda. The funder had no role in the design, conduct, analysis, interpretation, or writing of the study, and holds no commercial or financial interest in its findings or outcomes.

## Ethics Statement

Institutional Review Board Statement: The study was approved by the Ethics Committees of the Local Health Unit of Alto Alentejo (ULSAALE)–N.60/2024, 4th December and Nova Medical School, 24th April 2025.

## Consent

Informed consent was obtained from all subjects involved in the study. Written informed consent has been obtained from the patient (s) to publish this paper.

## Conflicts of Interest

The authors declare no conflicts of interest.

## Supporting information


**Data S1:** Supporting Information.

**Data S2:** Supporting Information.

**Data S3:** Supporting Information.

**Data S4:** Supporting Information.

**Data S5:** Supporting Information.

**Data S6:** Supporting Information.

**Data S7:** Supporting Information.

**Data S8:** Supporting Information.

**Data S9:** Supporting Information.

**Data S10:** Supporting Information.

**Data S11:** Supporting Information.

**Data S12:** Supporting Information.

**Data S13:** Supporting Information.

**Data S14:** Supporting Information.

**Data S15:** Supporting Information.

**Data S16:** Supporting Information.

**Data S17:** Supporting Information.

**Data S18:** Supporting Information.

**Data S19:** Supporting Information.

**Data S20:** Supporting Information.

**Data S21:** Supporting Information.

## Data Availability

The data that support the findings of this study are available from the corresponding author upon reasonable request.
