# Correction to “Multigene Germline Panel Testing in Gastric Cancer Patients in a Portuguese Population”

**DOI:** 10.1002/cam4.71826

**Published:** 2026-04-09

B. Mourato, B. Cordeiro, F. Costa Pinto, et al., “Multigene Germline Panel Testing in Gastric Cancer Patients in a Portuguese Population,” *Cancer Medicine* 15, no. 3 (2026): e71732, https://doi.org/10.1002/cam4.71732.

In the published version of this article, the author name **H. Capote** was inadvertently omitted from the author byline.

The correct author list and affiliations are provided below:

B. Mourato^1,2^| B. Cordeiro^2^| F. Costa Pinto^2^| N. Pratas^2^| H. Capote^2^| I. Fronteira^2^| M. Areia^3,4^ | R. Dinis^5^

^1^NOVA National School of Public Health, Public Health Research Centre, Comprehensive Health Research Center, CHRC, LA‐REAL, CCAL, NOVA University Lisbon, Lisbon, Portugal, Lisbon, Portugal

^2^Unidade Local de Saúde Do Alto Alentejo, Hospital Doutor José Maria Grande, Portalegre, Portugal

^3^Instituto Português De Oncologia De Coimbra, Coimbra, Portugal

^4^RISE@CI‐IPO (Health Research Network), Portuguese Oncology Institute of Porto (IPO Porto), Porto, Portugal

^5^Hospital Do Espírito Santo De Évora, Évora, Portugal

We apologize for this error.

