# Supplementary material for: Multigene Germline Panel Testing in Gastric Cancer Patients in a Portuguese Population
Source: Cancer Med. 2026 Mar 19;15(3):e71732. doi: 10.1002/cam4.71732 (PMC13093424; doi:10.1002/cam4.71732)
Supplement: Supplementary file 1 — Data S1: Supporting Information. [file CAM4-15-e71732-s010.docx]

**1. Summary Table: 15-Gene Panel (Hereditary Gastric Cancer Risk)**

This panel searches for point mutations and small deletions/duplications via Next Generation Sequencing (NGS) in 15 genes associated with hereditary gastric cancer risk. Multiplex Ligation-dependent Probe Amplification (MLPA) is used specifically for large rearrangements in the *CDH1* gene.

| **Gene (HGNC Symbol)** | **OMIM ID** | **Primary Analysis (NGS)** | **Supplementary Analysis (MLPA)** |
| --- | --- | --- | --- |
| **APC** | 611731 | Exons and Intron-Exon Junctions | Not Specified |
| **ATM** | 607585 | Exons and Intron-Exon Junctions | Not Specified |
| **BLM** | 604610 | Exons and Intron-Exon Junctions | Not Specified |
| **BMPR1A** | 601299 | Exons and Intron-Exon Junctions | Not Specified |
| **CDH1** | 192090 | Exons and Intron-Exon Junctions | Large Rearrangements |
| **CTNNA1** | 116805 | Exons and Intron-Exon Junctions | Not Specified |
| **EPCAM** | 185535 | Exons and Intron-Exon Junctions | Not Specified |
| **MLH1** | 120436 | Exons and Intron-Exon Junctions | Not Specified |
| **MSH2** | 609309 | Exons and Intron-Exon Junctions | Not Specified |
| **MSH6** | 600678 | Exons and Intron-Exon Junctions | Not Specified |
| **PMS2** | 600259 | Exons and Intron-Exon Junctions | Not Specified |
| **PTEN** | 601728 | Exons and Intron-Exon Junctions | Not Specified |
| **SMAD4** | 600993 | Exons and Intron-Exon Junctions | Not Specified |
| **STK11** | 602216 | Exons and Intron-Exon Junctions | Not Specified |
| **TP53** | 191170 | Exons and Intron-Exon Junctions | Not Specified |

**2. Summary Table: 30-Gene Panel (Hereditary Breast and Ovarian Cancer Risk)**

This panel uses NGS to search for point mutations and small indels in 30 genes. MLPA is used for large deletions/duplications in *BRCA1*, *BRCA2*, and *PALB2*, and for the Portuguese founder mutation in *BRCA2*.

| **Gene (HGNC Symbol)** | **OMIM ID** | **Primary Analysis (NGS)** | **Supplementary Analysis (MLPA)** |
| --- | --- | --- | --- |
| **ATM** | 607585 | Exons and Intron-Exon Junctions | Not Specified |
| **BARD1** | 601593 | Exons and Intron-Exon Junctions | Not Specified |
| **BLM** | 604610 | Exons and Intron-Exon Junctions | Not Specified |
| **BRCA1** | 113705 | Exons and Intron-Exon Junctions | Large Rearrangements |
| **BRCA2** | 600185 | Exons and Intron-Exon Junctions | Large Rearrangements & Portuguese Founder Mutation (c.156_157_insAlu) |
| **BRIP1** | 605882 | Exons and Intron-Exon Junctions | Not Specified |
| **CDH1** | 192090 | Exons and Intron-Exon Junctions | Not Specified |
| **CHEK2** | 604373 | Exons and Intron-Exon Junctions | Not Specified |
| **EPCAM** | 185535 | Exons and Intron-Exon Junctions | Not Specified |
| **FANCC** | 613899 | Exons and Intron-Exon Junctions | Not Specified |
| **MLH1** | 120436 | Exons and Intron-Exon Junctions | Not Specified |
| **MRE11A** | 600814 | Exons and Intron-Exon Junctions | Not Specified |
| **MSH2** | 609309 | Exons and Intron-Exon Junctions | Not Specified |
| **MSH6** | 600678 | Exons and Intron-Exon Junctions | Not Specified |
| **MUTYH** | 604933 | Exons and Intron-Exon Junctions | Not Specified |
| **NBN** | 602667 | Exons and Intron-Exon Junctions | Not Specified |
| **NF1** | 613113 | Exons and Intron-Exon Junctions | Not Specified |
| **PALB2** | 610355 | Exons and Intron-Exon Junctions | Large Rearrangements |
| **PMS2** | 600259 | Exons and Intron-Exon Junctions | Not Specified |
| **PTEN** | 601728 | Exons and Intron-Exon Junctions | Not Specified |
| **RAD50** | 604040 | Exons and Intron-Exon Junctions | Not Specified |
| **RAD51C** | 602774 | Exons and Intron-Exon Junctions | Not Specified |
| **RAD51D** | 602954 | Exons and Intron-Exon Junctions | Not Specified |
| **RECQL4** | 603780 | Exons and Intron-Exon Junctions | Not Specified |
| **RINT1** | 610089 | Exons and Intron-Exon Junctions | Not Specified |
| **SLX4** | 613278 | Exons and Intron-Exon Junctions | Not Specified |
| **SMARCA4** | 603254 | Exons and Intron-Exon Junctions | Not Specified |
| **STK11** | 602216 | Exons and Intron-Exon Junctions | Not Specified |
| **TP53** | 191170 | Exons and Intron-Exon Junctions | Not Specified |
| **XRCC2** | 600375 | Exons and Intron-Exon Junctions | Not Specified |

**3. Technical and Scientific Summary of Genetic Testing Procedure**

The genetic study involves the isolation of **genomic DNA from peripheral blood cells** and subsequent analysis using NGS and MLPA methodologies.

**Methodology Overview**

**A. Next Generation Sequencing (NGS)** NGS is the primary method used to detect **point mutations and small deletions/duplications** (indels).

1. **Scope:** Analysis covers **all exons** and **intron-exon transitions** (minimum of +/- 5pb) of the respective 15 or 30 genes.
2. **Platform and Kits:** Sequencing is executed on the **IonTorrent (S5) platform**. The process is **Amplicon based**, utilizing the **Ion AmpliSeq™ On-Demand Panel** and the **Ion AmpliSeq™ Library Kit** (2.0 or Plus). Library preparation uses the Ion 510 & Ion 520 & Ion 530 Kit-Chef.
3. **Quality Control:** Stringent metrics are applied, ensuring a **mean coverage depth superior to 250x** (often significantly higher, such as 652x or 1111x), and a **minimum 20x coverage superior to 98.5%**.

**B. Multiplex Ligation-dependent Probe Amplification (MLPA)** MLPA is a supplementary method used to identify **large rearrangements, deletions, and/or duplications**.

1. **Targets:**
   - **15-Gene Panel:** MLPA® Salsa® P083 CDH1 probes are used for *CDH1* large rearrangements.
   - **30-Gene Panel:** MLPA is applied to *BRCA1* (P002), *BRCA2* (P090), and *PALB2* (P260).
2. **Specific Screening:** The MLPA P090-BRCA2 panel is used to screen for the **Portuguese founder mutation**(**c.156_157 insertion Alu**) in the *BRCA2* gene.
3. **Detection:** Fragment analysis is performed by **capillary electrophoresis** (3130XL Genetic Analyzer, Applied Biosystems®) and analyzed using the **Coffalyser.net** software.

**Data Interpretation and Validation**

1. **Reference and Annotation:** Variant annotation is based on the **GRCh37 human genome reference version**. Interpretation utilizes data from databases such as **ClinVar** (2019-12), **Varsome** (2019-12), **dbSNP** (152), and EBI Variation HomoSapiens (91-37).
2. **Classification:** Mutational effects are predicted using algorithms including **PolyPhen, SIFT, LoF, Condel, BLOSUM62, CAROL, and fathmm-MKL**. Final classification adheres to the recommendations of the **American College of Medical Genetics and Genomics (ACMG)**.
3. **Reporting:** Only variants classified as **pathogenic, likely pathogenic, or variants of uncertain clinical significance (VUS)** are reported. The nomenclature follows the criteria established by the **Human Genome Variation Society (HGVS)**.
4. **Confirmation:**
   - All reported **pathogenic or likely pathogenic** variants occurring in coding regions with frequencies greater than 30% are **confirmed by Sanger sequencing**.
   - Positive MLPA results for *BRCA1* and *BRCA2* rearrangements are confirmed using specific confirmation probe panels (P087 and P077, respectively).
   - Positive results for the *BRCA2* Alu insertion are validated by **Polymerase Chain Reaction (PCR)**technology.

**Technical Limitations**

The testing procedure has technical limitations, including:

- The test **does not exclude pathogenic variants outside the sequenced regions**, specifically regulatory regions upstream or in deep introns (beyond +/- 5pb).
- It **does not detect variants below the technical detection limit (5%)**.
- The possibility of variants in complex genomic regions, highly repetitive sequences, or high **GC content regions**cannot be entirely excluded.
- The test **does not exclude variants in other genes** not included in the respective 15- or 30-gene panels.
- It does not exclude the possibility of other types of variants, such as large chromosomal rearrangements, if they are not specifically targeted by MLPA.
