# Supplementary material for: Multigene Germline Panel Testing in Gastric Cancer Patients in a Portuguese Population
Source: Cancer Med. 2026 Mar 19;15(3):e71732. doi: 10.1002/cam4.71732 (PMC13093424; doi:10.1002/cam4.71732)
Supplement: Supplementary file 2 — Data S2: Supporting Information. [file CAM4-15-e71732-s007.pdf]

02 - Dataset SPSS

Ageatdiagnose	Sex	BMI	OncologicalDiseases	Any_desease	CardiovascularDiseases	PulmonaryDiseases	PsychiatricNeurologicDisorders	GastrointestinalDisorders	ReproductiveSystemDisorders	OsteoarticularRheumatologicDiseases	AlcoholorTobaccoDependence	othercancersinthesamepatient	Ifsowhichcancer	Ageatdiagnoseofothercancer
63	F	25.3154542937384	2	1	2	2	2	2	1	2	2	2		
57	M	21.7738423573813	2	1	2	2	1	2	2	2	2	2		
84	M	32.4218339693988	2	1	1	2	2	2	1	2	2	2		
80	F	27.5312294543064	2	1	1	2	2	2	2	1	2	2		
79	F	27.0551508844953	2	1	1	2	2	2	2	1	2	2		
44	M	27.4048442906574	1	2	2	2	2	2	2	2	2	1	Colon	45
55	M	21.0771952275208	2	2	2	2	2	2	2	2	1	2		
84	F	22.2136784997223	2	1	1	2	2	1	2	2	2	2		
80	M	28.0102237316621	2	1	1	2	1	2	2	2	2	2		
88	F	12.5385802469136	2	2	2	2	2	2	2	2	2	2		
79	M	26.3575108997226	2	1	1	2	2	1	2	2	2	2		
55	M	17.3744528717966	2	1	2	2	1	2	2	2	2	2		
52	M	27.681660899654	1	1	1	2	2	2	2	2	2	1	Prostate	85
63	F	22.3289132754153	1	1	2	2	2	2	2	1	2	1	Breast	50
77	M	23.1404958677686	2	1	1	2	2	2	2	2	2	2		
82	M	27.681660899654	2	1	1	2	2	2	1	2	2	2		
82	M	22.77318640955	2	1	2	2	2	1	2	2	2	2		
64	M	32.1799307958478	2	1	1	1	1	2	2	2	2	2		
75	M	30.1038062283737	2	1	1	2	2	2	1	2	2	2		
84	M	23.2550186382135	1	1	1	2	1	2	2	2	2	1	Colon	80
71	M	19.4150094353317	2	1	1	2	1	2	2	2	2	2		
88	F	18.7304890738814	2	1	1	2	1	2	2	1	2	2		
69	M	24.6464646464646	1	1	1	1	1	2	2	2	2	1	Meningioma	
65	M	27.8520079792239	1	1	1	2	2	1	2	2	2	1	Protate	64
32	M	24.9770431588613	2	1	2	2	1	1	2	2	2	2		
66	M	24.7675659198293	2	1	1	2	2	2	2	1	2	2		
44	M	24.092970521542	2	2	2	2	2	2	2	2	2	2		
76	M	24.092970521542	1	1	1	2	2	2	2	2	2	1	Kidney	67
79	M	29.0478315550327	2	1	1	1	2	2	2	2	2	2		
74	M	29.5133545364386	2	1	1	2	2	2	2	1	2	2		
79	M	27.5802003193497	2	1	1	2	2	2	2	2	2	2		
80	M	26.291723822588	1	1	1	2	2	2	2	2	2	1	Protate	68
49	M	22.038567493113	2	1	2	2	2	2	2	1	1	2		
66	M	22.530612244898	2	1	2	2	1	2	2	2	2	2		
76	F	31.1634349030471	2	1	1	2	2	2	2	1	2	2		
79	M	25.0761930481077	2	1	1	1	2	2	2	2	2	2		
91	F	22.6666666666667	2	1	1	2	2	2	2	2	2	2		
55	F	31.25	2	1	1	2	1	2	2	2	2	2		
68	M	31.7739318550568	1	1	1	2	2	1	2	2	2	1	Prostate	63
71	M	19.5716812826979	2	2	2	2	2	2	2	2	2	2		
71	F	32.9506802721088	1	1	1	2	1	2	2	2	2	1	Thyroid	71
66	F	25.7777777777778	1	1	1	2	1	2	2	2	2	1	Colon	66
80	M	19.0039083509627	2	1	1	2	2	1	2	1	2	2		
85	F	16.2278388575601	2	1	1	2	2	1	1	2	2	2		
75	M	24.2214532871972	2	1	2	1	2	2	2	2	1	2		
70	M	18.7783446712018	2	1	1	2	2	1	2	2	2	2		
87	M	20.6611570247934	2	1	2	2	2	1	2	2	2	2		
63	M	24.9770431588613	2	1	1	2	2	2	1	2	2	2		
55	F	26.7759341149742	2	1	1	2	2	1	2	2	2	2		
79	F	20.0288415318058	2	1	1	2	2	2	2	1	2	2		
87	M	25.0657462195924	2	1	1	2	2	2	1	2	2	2		

Directrelativesdiagnosedwithcancer	More_than_1_relative	Famlial_Stomach_cancer	Sutype_ADC	Relative1typeofrelationship	Relative1AgeofDiagnostic	Relative1Organsystemafected	Relative2typeofrelationship	Relative2AgeofDiagnostic	Relative2Organsystemafected	Relative3typeofrelationship	Relative3AgeofDiagnostic
1	1	2	Diffuse	Sister	40 (death)	Ovary	Brother	71 (death)	Testis	Sister	71
1	1	1	Intestinal	Uncle	75	Stomach	Grandfather	80	Prostate	Aunt	.
1	1	2	Intestinal	Father	89 (death)	Prostate	Brother	70 (death)	Prostate		
1	2	2	Intestinal	Brother	76	Lung					
1	1	1	Intestinal	Brother	67	Lung	Sister	77	Stomach	Father	70
1	2	2	Intestinal	Father	56	Lung					
2	2	2	Intestinal								
1	2	2	Mixed	Father	72 (death)	Lung					
2	2	2	Intestinal								
2	2	2	Intestinal								
2	2	2	Intestinal								
2	2	2	Diffuse								
1	1	1	Intestinal	Brother	45	Stomach	Niece	Unknown	Stomach	Sister	Unknown
1	1	1	Diffuse	Mother	52	Breast	Father	Unknown	Stomach	Grandmother	Unknown
2	2	2	Intestinal								
1	2	2	Diffuse	Brother	50	Colon					
2	2	2	Intestinal								
1	1	1	Intestinal	Mother	96	Stomach	Cousin	Unknown	Stomach		
2	2	2	Intestinal								
2	2	2	Intestinal	Father	50	Lung					
2	2	2	Intestinal								
2	2	2	Intestinal								
1	1	2	Intestinal	Father	85	Prostate	Mother	90	Colon	8 Uncles (4 from mother and 4 from fahter)	Digestive cancers
1	2	1	Intestinal	Cousin	72	Stomach					
2	2	2	Mixed								
1	2	2	Diffuse	Mother	?	Kidney and leucemia					
1	2	2	Mixed	Brother	45	Colon					
2	2	2	Intestinal								
1	1	2	Intestinal	Father	77	Breast	Brother	70 (death)	Prostate	Mother	80
2	2	2	Intestinal								
2	2	2	Intestinal								
2	2	2	Intestinal								
1	2	2	Intestinal	Grandparents	?	.					
1	1	2	Diffuse	Cousins	50	Colon	Cousin	33 (death)	Colon		
1	1	1	Intestinal	Sister	81	Stomach	Father	70	Stomach		
1	1	1	Intestinal	Father	80	Colon	Brother	74	Stomach	Mother	79
1	1	2	Intestinal	Sister	60	Breast	Sister	70	Ovary	Father	83
1	2	2	Intestinal	Daughter	34	Cervix					
2	2	2	Diffuse								
1	1	2	Intestinal	Brother	62	Prostate	Sister	58	Lymphoproliferative disease		
1	2	2	Intestinal	Mother	57	Breast					
1	2	2	Diffuse	Brother	71	Rectum					
1	1	2	Intestinal	Sister	58	Breast	Mother	79	Skin		
1	1	1	Mixed	Aunt	70	Colon	Father	82	Stomach		
1	2	2	Intestinal	Mother	58	Colon					
1	2	2	Diffuse	Brother	65	Skin					
1	1	1	Intestinal	Mother	55	Stomach	Brother	80	Prostate		
2	2	2	Intestinal								
2	2	2	Diffuse								
2	2	2	Intestinal								
1	1	1	Intestinal	Father	85	Stomach	Mother	87	Skin		

Relative3Organsystemafected	Notes	Tumourlocation	Histology	Histology_resume	cTNM	Clinical_Stage	@1stCDTdecision	Peri_Op_QT	Palliative_QT	Surgery	STG	TG	TypeofSurgery	TNMpostop	p_Stage
Skin	Nephew with testicular cancer	Body	ADC Diffuse, Signet cells	Diffuse	T2-3N1M0	III	QT peri-op -> Surgery	1	2	1	1		Laparoscopy converted, Subtotal gastrectomy, D2, Roux Y	ypT3N3b(16/48)M0	IIIC
Lung	Nephew with oral cancer	Body, Antrum	ADC Intestinal	Intestinal	T4N2M0	III	QT peri-op -> Surgery	1	2	1	1		Laparoscopy, Subtotal gastrectomy, D1+, Roux Y	pT4aN0(34)M0	IIB
		Incisura	ADC Intestinal	Intestinal	T2N0M0	I	QT peri-op -> Surgery	1	2	1	1		Laparoscopy converted, Subtotal gastrectomy, D1+, Bilroth I	ypT1N0(10)M0	IA
		Incisura	ADC Intestinal	Intestinal	T2N1-2M0	IIA	QT peri-op -> Surgery	1	2	1		1	Laparoscopy converted, Total gastrectomy, D2, Roux Y	ypT0N0(15)M0	0
Stomach		Antrum	ADC Intestinal	Intestinal	T2N1-2/M0	IIA	QT peri-op -> Surgery	1	2	1	1		Laparoscopy converted, Total gastrectomy, D2, Roux Y	ypT1N0(16)M0	IA
		Body, Antrum	ADC Intestinal	Intestinal	T2N2M0	IIA	QT peri-op -> Surgery	1	2	1	1		Laparoscopy, Subtotal gastrectomy, D2, Roux Y	ypT3N0(26)M0	IIA
		Cardia	ADC Intestinal	Intestinal	T3N+M1	IVB	QT peri-op	1	2				No surgery	.	
		Antrum	ADC Intestinal, ADC Diffuse, Signet cells	Mixed	T2N0M0	I	QT peri-op → Not tolerated → Surgery	1	2	1	1		Laparoscopy, Subtotal gastrectomy, D1+, Bilroth I	ypTisN0(27)M0	0
		Pyloros	ADC Intestinal	Intestinal	T2N0M0	I	Surgery	2	2	1	1		Laparoscopy, Subtotal gastrectomy, Roux Y	pT2N0M0	IB
		Antrum	ADC Intestinal	Intestinal	T3N1M0	III	Hemostatic RT -> Surgery	2	2	1	1		Laparotomy, Subtotal gastrectomy, Bilroth I	ypT3N1(19)M0	IIB
		Body	ADC Intestinal	Intestinal	T3N1M1 (lung)	IVB	QT peri-op -> Surgery	1	2	1		1	Laparoscopy, Total gastrectomy, Roux Y	ypT0N0Mx	0
		Incisura	ADC Diffuse	Diffuse	T2N1M0	IIA	QT peri-op -> Surgery	1	2	1	1		Laparoscopy, D2, Roux Y	ypT1bN3a(11/27)M0	IIB
Breast		Unknown	ADC Intestinal	Intestinal	Unknwon		Unknown	2	2	1		1	Total gastrectomy	.	
Stomach and pancreas	Grandmother: Breast ; Cousins: breast and prostate	Body, Antrum	ADC Diffuse	Diffuse	T3N1M1	IVB	Palliative QT	2	1				No surgery	.	
		Antrum	ADC Intestinal	Intestinal	T3N1M0	III	QT peri-op -> Surgery	1	2	1	1		Laparoscopy, Subtotal gastrectomy, Roux Y	cT2N1(21)M0	IIA
		Antrum	ADC Diffuse	Diffuse	T1bNxM0	IIA	Surgery	2	2	1	1		Laparotomy, Subtotal gastrectomy, D2, Roux Y	pT1N0Mx	IA
		Body	ADC Intestinal	Intestinal	T1bN0M0	I	Surgery	2	2	1	1		Laparoscopy, Subtotal gastrectomy, D2, Roux Y	pT1N0(30)M0	IA
		Fundus	ADC Intestinal	Intestinal	T2N1M0	IIA	QT peri-op -> Surgery	1	2	1		1	Total gastrectomy	pT2N0	IB
		Antrum	ADC Intestinal	Intestinal	T4N1M0	III	QT peri-op -> Surgery	1	2	1	1		Subtotal gastrectomy, Bilroth II	ypT4a N0 Mx	IIB
		Body	ADC Intestinal	Intestinal	T3N2M1	IVB	Palliative QT	2	1				No surgery	.	
		Antrum	ADC Intestinal	Intestinal	T4N0M0	III	Surgery	2	2	1	1		Laparoscopy, Subtotal gastrectomy, D2, Bilroth I	pT4aN2M1	IV
		Body, Antrum	ADC Intestinal	Intestinal	T1N0M0	I	Surgery	2	2	1	1		Laparoscopy, Subtotal gastrectomy, D1+, Bilroth I	pT1N1(18)M0	IB
		Antrum	ADC Intestinal	Intestinal	T4bN2M1	IVB	QT peri-op -> Palliative	2	1				No surgery	.	
		Body	ADC Intestinal	Intestinal	T4aN3bM1	IVB	Palliative QT	2	1				No surgery	.	
		Pyloros	ADC Intestinal, ADC Diffuse, Signet cells	Mixed	T3N2M1	IVB	Palliative QT	2	1				No surgery	pT3N2M1	IV
		Body	ADC Diffuse, Signet cells	Diffuse	T2N0M0	I	QT peri-op -> Surgery	1	2	1	1		Laparoscopy, Subtotal gastrectomy, D2, Roux Y	ypT3 N0(24) M0	IIA
		Pyloros	ADC Intestinal, ADC Diffuse, Signet cells	Mixed	T3N1M1	IVB	Surgery -> Palliative QT	2	1	1	1		Laparotomy, Roux Y	pT3 N2(6/10) M1	IIIA
		Body	ADC Intestinal	Intestinal	T2N0M0	I	QT peri-op -> Surgery	1	2	1	1		Laparoscopy, Subtotal gastrectomy, D2, Bilroth II	ypT2 pN1(15) pM	IIA
Colon		Cardia, Fundus	ADC Intestinal	Intestinal	T2N2M0	IIA	Palliative QT	2	1				No surgery	.	
		Cardia	ADC Intestinal	Intestinal	T4aN+M0	III	Palliative QT	2	1				No surgery	.	
		Body, Antrum	ADC Intestinal	Intestinal	T2N0M0	I	Surgery	2	2	1	1		Laparotomy, Subtotal gastrectomy, D1+, Bilroth I	T2N0M0	IB
		Antrum	ADC Intestinal	Intestinal	T3-4aN+M0	III	QT peri-op -> Surgery	1	2	1	1		Laparoscopy, Subtotal gastrectomy, D2, Roux Y	pT2 pN0(19) pM0	IB
		Cardia	ADC Intestinal	Intestinal	T2-3N2M1	IVB	Palliative QT	2	1				No surgery	.	
		Body, Antrum	ADC Diffuse, Signet cells	Diffuse	T2N1-2M0	IIA	QT peri-op -> Surgery	1	2	1	1		Laparoscopy, Subtotal gastrectomy, D2, Roux Y	.	
		Incisura	ADC Intestinal	Intestinal	T2N0M0	I	QT peri-op -> Surgery	1	2	1	1		Laparoscopy, Subtotal gastrectomy, D2, Roux Y	ypT3N0(15)M0	IIA
Breast		Body	ADC Intestinal	Intestinal	T2N2M0	I	Palliative QT	2	1				No surgery	.	
Oesophagus	daughter: pulmonary thromboembolism	Antrum	HGIN	Intestinal	T1-2N0M0	I	Surgery	2	2	1	1		Laparoscopy, Subtotal gastrectomy, D1+, Bilroth I	pT1bN1(8)M0	IB
		Antrum	ADC Intestinal	Intestinal	-		Surgery	2	2	1	1		Laparotomy, Subtotal gastrectomy, D2, Bilroth II	pT2N0M0	IB
		Antrum	ADC Diffuse	Diffuse	T1-2N0M0	I	Surgery	2	2	1	1		Laparoscopy, Subtotal gastrectomy, D1+, Roux Y	pT2N0(10)M0	IB
		Body, Antrum	ADC Intestinal	Intestinal	T3N0M0	IIB	Surgery	2	2	1	1		Laparoscopy, Subtotal gastrectomy, D2, Roux Y	pT3N0(22)M0	IIA
		Body	ADC Intestinal	Intestinal	T4aN0M0	IIB	QT peri-op -> Surgery	1	2	1	1		Laparoscopy, Subtotal gastrectomy, D2, Roux Y	pT4aN2(18)M0	IIIA
		Body, Antrum	ADC Diffuse	Diffuse	T3N0M0	IIB	QT peri-op -> Surgery	1	2	1	1		Subtotal gastrectomy, D2, Roux Y	pT3N1(30)M0	IIB
		Pyloros	HGIN	Intestinal	T2N0M0	I	Surgery	2	2	1	1		Laparoscopy, Subtotal gastrectomy, D1+, Roux Y	pT3N0(9)M0	IIA
		Incisura	ADC Intestinal, Signet cells	Mixed	T2N0M0	I	QT peri-op -> Surgery	1	2	1	1		Laparoscopy, Subtotal gastrectomy, Bilroth I	ypT0N0M0	0
		Body	ADC Intestinal	Intestinal	T4N0M0	III	QT peri-op -> Surgery	1	2	1	1		Laparoscopy, D2, Roux Y	ypT1bN0(10)M0	IA
		Cardia, Body, Antrum	ADC Diffuse, Signet cells	Diffuse	T3/4N1M0	III	QT peri-op -> Surgery	1	2	1		1	Laparoscopy, Total gastrectomy, D2, Roux Y	ypT3N0(16)M0	IIA
		Antrum	ADC Intestinal	Intestinal	T2N1M0	IIA	QT peri-op -> Surgery	1	2	1	1		Laparoscopy, Subtotal gastrectomy, D2, Roux Y	ypT2N0(22)M0	IB
		Body, Antrum	ADC Intestinal	Intestinal	T1N0M0	I	Surgery	2	2	1	1		Laparoscopy, Subtotal gastrectomy, Roux Y	pT1N0(20)M0	IA
		Antrum, Pyloros	ADC Diffuse	Diffuse	T2N0M0	I	Surgery	2	2	1	1		Laparoscopy, Subtotal gastrectomy, D1+, Bilroth I	pT3N1(8)M0	IIB
		Body	ADC Intestinal	Intestinal	T2-3N0M0	IIB	QT peri-op -> Surgery	1	2	1	1		Laparoscopy, Subtotal gastrectomy, D2, Roux Y	ypT3N0(9)M0	IIA
		Body	ADC Intestinal	Intestinal	T2-3N+M0	III	QT peri-op -> Surgery	1	2	1	1		Laparoscopy, Subtotal gastrectomy, D1+, Roux Y	ypT1N0(10)M0	IA

TypeofMGPT	MGPT_Mutations_VUS	MGPT_Mutations	PV	LP	VUS	QT	@12monthsurvival	Years_since_diagnostic	Deathdate
4013 - 30 genes	2	2	.	.	.	1	1	3	
4013 - 30 genes	2	2	.	.	.	1	1	3	
4005 - 15 genes	2	2	.	.	.	1	1	3	
4005 - 15 genes	2	2	.	.	.	1	1	2	
4005 - 15 genes	2	2	.	.	.	1	1	3	
4013 - 30 genes	1	1	MLH1 c1409+2T>G	.	.	1	1	2	
4005 - 15 genes	2	2	.	.	.	1	1	2	
4005 - 15 genes	2	2	.	.	.	2	1	2	
4005 - 15 genes	2	2	.	.	.	2	1	5	
4005 - 15 genes	2	2	.	.	.	2	1	1	12/5/2024
4005 - 15 genes	2	2	.	.	.	1	1	2	
4013 - 30 genes	1	2	.	.	BLM	1	1	2	
4013 - 30 genes	1	1	.	Gene PMS2 c.1204C>T p.(Gln402Ter)	Gene MSH6 c.3452C>G p.(Ala1151Gly)	2	1	3	
4005 - 15 genes	2	2	.	.	.	2	2	0	8/1/2023
4005 - 15 genes	1	1	MSH6 c.3261del.p	.	.	1	1	2	
4005 - 15 genes	2	2	.	.	.	2	1	5	
4005 - 15 genes	2	2	.	.	.	2	1	1	
4005 - 15 genes	2	2	.	.	.	1	1	3	
4005 - 15 genes	2	2	.	.	.	2	2	0	7/25/2024
4005 - 15 genes	2	2	.	.	.	1	2	0	1/20/2025
4005 - 15 genes	2	2	.	.	.	1	1	1	
4005 - 15 genes	1	2	.	.	ATM: c.4414T>Gp.(Leu1472Val)	2	1	1	
4005 - 15 genes	2	2	.	.	.	1	1	1	6/29/2025
4005 - 15 genes	2	2	.	.	.	1	1	1	11/7/2024
4013 - 30 genes	1	1	.	MLH1	.	1	2	0	7/28/2022
4013 - 30 genes	2	2	.	.	.	1	1	1	
4013 - 30 genes	1	1	CHEK2 c.592+3A>T.	.	.	2	2	0	7/28/2022
4005 - 15 genes	2	2	.	.	.	1	0	1	
4013 - 30 genes	2	2	.	.	.	1	0	1	
4013 - 30 genes	2	2	.	.	.	1	0	0	
4013 - 30 genes	1	2	.	.	c.3542A>G p.(Asp1181Gly) 2 gene MSH6	2	0	0	
4013 - 30 genes	2	2	.	.	.	1	0	0	
4005 - 15 genes	2	2	.	.	.	1	1	1	
4005 - 15 genes	2	2	.	.	.	1	0	0	
4013 - 30 genes	2	2	.	.	.	1	0	0	
4013 - 30 genes	2	2	.	.	.	1	0	0	
4005 - 15 genes	2	2	.	.	.	2	1	4	
4005 - 15 genes	2	2	.	.	.	2	1	2	
4005 - 15 genes	1	2	.	.	3: ATM; TP53; BLM	1	1	5	
4005 - 15 genes	2	2	.	.	.	1	1	5	
4005 - 15 genes	2	2	.	.	.	1	1	5	
4013 - 30 genes	1	1	2: BLM c2206dup	.	.	1	1	5	
4005 - 15 genes	2	2	.	.	.	2	1	5	
4005 - 15 genes	2	2	.	.	.	1	1	5	
4005 - 15 genes	2	2	.	.	.	1	1	4	
4005 - 15 genes	2	2	.	.	.	1	1	3	
4005 - 15 genes	2	2	.	.	.	1	1	3	
4005 - 15 genes	2	2	.	.	.	2	1	3	
4005 - 15 genes	1	2	.	.	MSH6	1	1	3	
4005 - 15 genes	2	2	.	.	.	1	1	3	
4005 - 15 genes	2	2	.	.	.	1	1	3	
