# Supplementary material for: Multigene Germline Panel Testing in Gastric Cancer Patients in a Portuguese Population
Source: Cancer Med. 2026 Mar 19;15(3):e71732. doi: 10.1002/cam4.71732 (PMC13093424; doi:10.1002/cam4.71732)

Statistics

Age at diagnose

N	Valid	51
	Missing	0
Mean		71.04
Std. Error of Mean		1.845
Median		75.00
Mode		79
Std. Deviation		13.176
Variance		173.598
Range		59
Minimum		32
Maximum		91
Sum		3623
Percentiles	25	63.00
	50	75.00
	75	80.00

Age at diagnose

		Frequency	Percent	Valid Percent	Cumulative Percent
Valid	32	1	2.0	2.0	2.0
	44	2	3.9	3.9	5.9

Age at diagnose

	Frequency	Percent	Valid Percent	Cumulative Percent
49	1	2.0	2.0	7.8
52	1	2.0	2.0	9.8
55	4	7.8	7.8	17.6
57	1	2.0	2.0	19.6
63	3	5.9	5.9	25.5
64	1	2.0	2.0	27.5
65	1	2.0	2.0	29.4
66	3	5.9	5.9	35.3
68	1	2.0	2.0	37.3
69	1	2.0	2.0	39.2
70	1	2.0	2.0	41.2
71	3	5.9	5.9	47.1
74	1	2.0	2.0	49.0
75	2	3.9	3.9	52.9
76	2	3.9	3.9	56.9
77	1	2.0	2.0	58.8
79	6	11.8	11.8	70.6
80	4	7.8	7.8	78.4
82	2	3.9	3.9	82.4
84	3	5.9	5.9	88.2
85	1	2.0	2.0	90.2
87	2	3.9	3.9	94.1
88	2	3.9	3.9	98.0
91	1	2.0	2.0	100.0
Total	51	100.0	100.0	

Age at diagnose

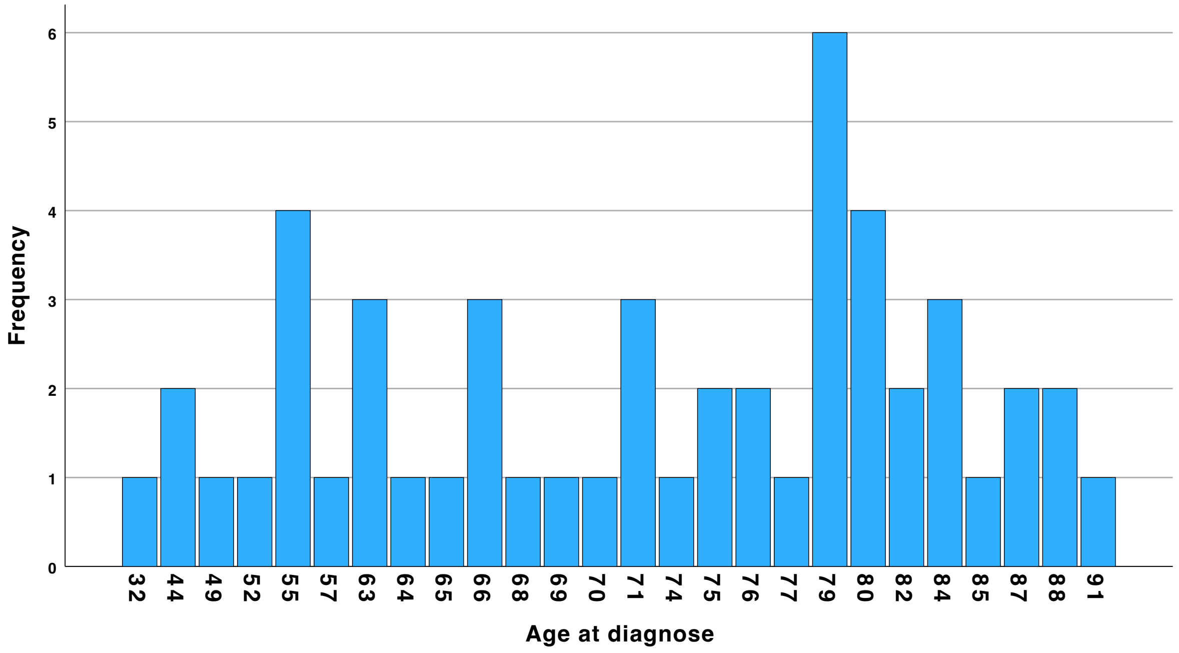

Supplement: Supplementary file 3 — Data S3: Supporting Information. [file CAM4-15-e71732-s016.pdf]
