# Supplementary material for: Multigene Germline Panel Testing in Gastric Cancer Patients in a Portuguese Population
Source: Cancer Med. 2026 Mar 19;15(3):e71732. doi: 10.1002/cam4.71732 (PMC13093424; doi:10.1002/cam4.71732)
Supplement: Supplementary file 4 — Data S4: Supporting Information. [file CAM4-15-e71732-s002.pdf]

BMI Statistics

Kg/m2

N	Valid	51
	Missing	0
Mean		24.7013
Std. Error of Mean		.62513
Median		24.9770
Mode		24.09[a]
Std. Deviation		4.46435
Variance		19.930
Minimum		12.54
Maximum		32.95
Sum		1259.77

a. Multiple modes exist. The smallest value is shown
