# Supplementary material for: Multigene Germline Panel Testing in Gastric Cancer Patients in a Portuguese Population
Source: Cancer Med. 2026 Mar 19;15(3):e71732. doi: 10.1002/cam4.71732 (PMC13093424; doi:10.1002/cam4.71732)
Supplement: Supplementary file 6 — Data S6: Supporting Information. [file CAM4-15-e71732-s017.pdf]

T-Test

Group Statistics

	PV or LP on MGPT	N	Mean	Std. Deviation	Std. Error Mean
Kg/m2	Yes	6	25.5125	1.80470	.73677
	No	45	24.5931	4.70918	.70200

Independent Samples Test

		Levene's Test for Equality of Variances		t-test for Equality of Means	
		F	Sig.	t	df
Kg/m2	Equal variances assumed	4.248	.045	.470	49
	Equal variances not assumed			.903	16.641

Independent Samples Test

		t-test for Equality of Means			
		Significance		Mean Difference	Std. Error Difference
		One-Sided p	Two-Sided p		
Kg/m2	Equal variances assumed	.320	.640	.91932	1.95556
	Equal variances not assumed	.190	.379	.91932	1.01766

Independent Samples Test

		t-test for Equality of Means	
		95% Confidence Interval of the Difference	
		Lower	Upper
Kg/m2	Equal variances assumed	-3.01052	4.84917
	Equal variances not assumed	-1.23129	3.06994

Independent Samples Effect Sizes

		Standardizer[a]	Point Estimate	95% Confidence Interval	
				Lower	Upper
Kg/m2	Cohen's d	4.49954	.204	-.650	1.056
	Hedges' correction	4.56990	.201	-.640	1.040
	Glass's delta	4.70918	.195	-.659	1.047

a. The denominator used in estimating the effect sizes.
Cohen's d uses the pooled standard deviation.
Hedges' correction uses the pooled standard deviation, plus a correction factor.
Glass's delta uses the sample standard deviation of the control (i.e., the …
