# Supplementary material for: Multigene Germline Panel Testing in Gastric Cancer Patients in a Portuguese Population
Source: Cancer Med. 2026 Mar 19;15(3):e71732. doi: 10.1002/cam4.71732 (PMC13093424; doi:10.1002/cam4.71732)
Supplement: Supplementary file 7 — Data S7: Supporting Information. [file CAM4-15-e71732-s003.pdf]

Sex * PV or LP on MGPT Crosstabulation

			PV or LP on MGPT		
			Yes	No	Total
Sex	F	Count	1	14	15
		% within PV or LP on MGPT	16.7%	31.1%	29.4%
	M	Count	5	31	36
		% within PV or LP on MGPT	83.3%	68.9%	70.6%
Total		Count	6	45	51
		% within PV or LP on MGPT	100.0%	100.0%	100.0%

Chi-Square Tests

	Value	df	Asymptotic Significance (2-sided)	Exact Sig. (2-sided)	Exact Sig. (1-sided)
Pearson Chi-Square	.532[a]	1	.466		
Continuity Correction[b]	.064	1	.801		
Likelihood Ratio	.586	1	.444		
Fisher's Exact Test				.657	.422
N of Valid Cases	51				

a. 2 cells (50.0%) have expected count less than 5. The minimum expected count is 1.76.

b. Computed only for a 2x2 table
