# Supplementary material for: Multigene Germline Panel Testing in Gastric Cancer Patients in a Portuguese Population
Source: Cancer Med. 2026 Mar 19;15(3):e71732. doi: 10.1002/cam4.71732 (PMC13093424; doi:10.1002/cam4.71732)
Supplement: Supplementary file 8 — Data S8: Supporting Information. [file CAM4-15-e71732-s020.pdf]

Descriptives

	PV or LP on MGPT			Statistic	Std. Error
Age at diagnose	Yes	Mean		52.50	6.702
		95% Confidence Interval for Mean	Lower Bound	35.27	
			Upper Bound	69.73	
		5% Trimmed Mean		52.28	
		Median		48.00	
		Variance		269.500	
		Std. Deviation		16.416	
		Minimum		32	
		Maximum		77	
		Range		45	
		Interquartile Range		28	
		Skewness		.497	.845
		Kurtosis		-.655	1.741
	No	Mean		73.51	1.588
		95% Confidence Interval for Mean	Lower Bound	70.31	
			Upper Bound	76.71	
		5% Trimmed Mean		73.81	
		Median		76.00	
		Variance		113.528	
		Std. Deviation		10.655	
		Minimum		49	
		Maximum		91	
		Range		42	
		Interquartile Range		16	
		Skewness		-.513	.354
		Kurtosis		-.614	.695

Age at diagnose

Stem-and-Leaf Plots

```
Age at diagnose Stem-and-Leaf Plot for
MGPT_Mutations= Yes

 Frequency    Stem &  Leaf

     3.00        0 .  344
     3.00        0 .  567

 Stem width:   **
 Each leaf:        1 case(s)



Age at diagnose Stem-and-Leaf Plot for
MGPT_Mutations= No

 Frequency    Stem &  Leaf

     1.00        4 .  9
```

```
     .00        5 .
    5.00        5 .  55557
    4.00        6 .  3334
    5.00        6 .  56689
    5.00        7 .  01114
   10.00        7 .  5566999999
    9.00        8 .  000022444
    5.00        8 .  57788
    1.00        9 .  1

Stem width:  10
Each leaf:        1 case(s)
```

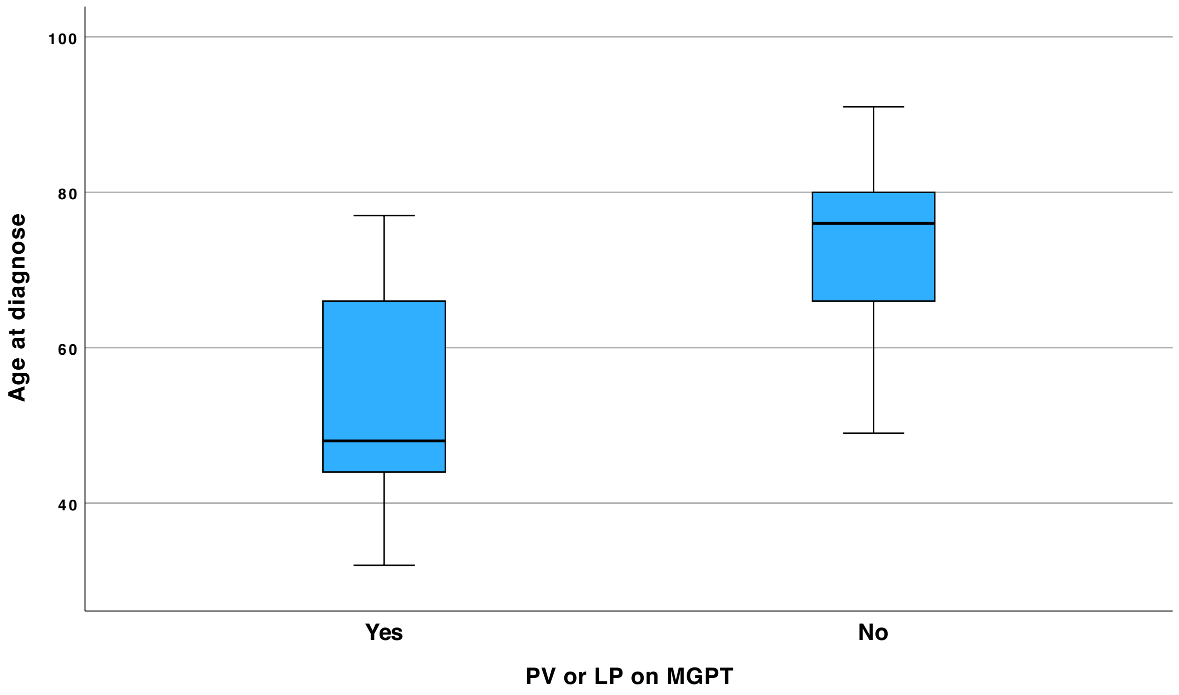

Mann-Whitney Test

Ranks

	PV or LP on MGPT	N	Mean Rank	Sum of Ranks
Age at diagnose	Yes	6	9.67	58.00
	No	45	28.18	1268.00
	Total	51		

Test Statistics[a]

	Age at diagnose
Mann-Whitney U	37.000
Wilcoxon W	58.000
Z	-2.870
Asymp. Sig. (2-tailed)	.004
Exact Sig. [2*(1-tailed Sig.)]	.002[b]

a. Grouping Variable: PV or LP on MGPT

b. Not corrected for ties.
