# Supplementary material for: Multigene Germline Panel Testing in Gastric Cancer Patients in a Portuguese Population
Source: Cancer Med. 2026 Mar 19;15(3):e71732. doi: 10.1002/cam4.71732 (PMC13093424; doi:10.1002/cam4.71732)
Supplement: Supplementary file 9 — Data S9: Supporting Information. [file CAM4-15-e71732-s001.pdf]

Case Processing Summary

	Cases					
	Valid		Missing		Total	
	N	Percent	N	Percent	N	Percent
Cardiovascular Diseases * PV or LP on MGPT	51	100.0%	0	0.0%	51	100.0%
Pulmonary Diseases * PV or LP on MGPT	51	100.0%	0	0.0%	51	100.0%
Psychiatric / Neurologic Disorders * PV or LP on MGPT	51	100.0%	0	0.0%	51	100.0%
Gastrointestinal Disorders * PV or LP on MGPT	51	100.0%	0	0.0%	51	100.0%
Reproductive System Disorders * PV or LP on MGPT	51	100.0%	0	0.0%	51	100.0%
Osteoarticular / Rheumatologic Diseases * PV or LP on MGPT	51	100.0%	0	0.0%	51	100.0%
Alcohol or Tobacco Dependence * PV or LP on MGPT	51	100.0%	0	0.0%	51	100.0%

Cardiovascular Diseases * PV or LP on MGPT

Crosstab

			PV or LP on MGPT		Total
			Yes	No	
Cardiovascular Diseases	Yes	Count	3	33	36
		% within PV or LP on MGPT	50.0%	73.3%	70.6%
	No	Count	3	12	15
		% within PV or LP on MGPT	50.0%	26.7%	29.4%
Total		Count	6	45	51
		% within PV or LP on MGPT	100.0%	100.0%	100.0%

Chi-Square Tests

	Value	df	Asymptotic Significance (2-sided)	Exact Sig. (2-sided)	Exact Sig. (1-sided)
Pearson Chi-Square	1.388[a]	1	.239		
Continuity Correction[b]	.492	1	.483		
Likelihood Ratio	1.281	1	.258		
Fisher's Exact Test				.343	.234
Linear-by-Linear Association	1.361	1	.243		
N of Valid Cases	51				

a. 2 cells (50.0%) have expected count less than 5. The minimum expected count is 1.76.

b. Computed only for a 2x2 table

Pulmonary Diseases * PV or LP on MGPT

Crosstab

			PV or LP on MGPT		
			Yes	No	Total
Pulmonary Diseases	Yes	Count	0	5	5
		% within PV or LP on MGPT	0.0%	11.1%	9.8%
	No	Count	6	40	46
		% within PV or LP on MGPT	100.0%	88.9%	90.2%
Total		Count	6	45	51
		% within PV or LP on MGPT	100.0%	100.0%	100.0%

Chi-Square Tests

	Value	df	Asymptotic Significance (2-sided)	Exact Sig. (2-sided)	Exact Sig. (1-sided)
Pearson Chi-Square	.739[a]	1	.390		
Continuity Correction[b]	.017	1	.897		
Likelihood Ratio	1.322	1	.250		
Fisher's Exact Test				1.000	.520
Linear-by-Linear Association	.725	1	.395		
N of Valid Cases	51				

a. 2 cells (50.0%) have expected count less than 5. The minimum expected count is .59.

b. Computed only for a 2x2 table

Psychiatric / Neurologic Disorders * PV or LP on MGPT

Crosstab

			PV or LP on MGPT		
			Yes	No	Total
Psychiatric / Neurologic Disorders	Yes	Count	2	11	13
		% within PV or LP on MGPT	33.3%	24.4%	25.5%
	No	Count	4	34	38
		% within PV or LP on MGPT	66.7%	75.6%	74.5%
Total		Count	6	45	51
		% within PV or LP on MGPT	100.0%	100.0%	100.0%

Chi-Square Tests

	Value	df	Asymptotic Significance (2-sided)	Exact Sig. (2-sided)	Exact Sig. (1-sided)
Pearson Chi-Square	.220[a]	1	.639		
Continuity Correction[b]	.000	1	1.000		
Likelihood Ratio	.209	1	.647		
Fisher's Exact Test				.638	.484
Linear-by-Linear Association	.216	1	.642		
N of Valid Cases	51				

a. 2 cells (50.0%) have expected count less than 5. The minimum expected count is 1.53.

b. Computed only for a 2x2 table

Gastrointestinal Disorders * PV or LP on MGPT

Crosstab

			PV or LP on MGPT		
			Yes	No	Total
Gastrointestinal Disorders	Yes	Count	1	10	11
		% within PV or LP on MGPT	16.7%	22.2%	21.6%
	No	Count	5	35	40
		% within PV or LP on MGPT	83.3%	77.8%	78.4%
Total		Count	6	45	51
		% within PV or LP on MGPT	100.0%	100.0%	100.0%

Chi-Square Tests

	Value	df	Asymptotic Significance (2-sided)	Exact Sig. (2-sided)	Exact Sig. (1-sided)
Pearson Chi-Square	.097[a]	1	.756		
Continuity Correction[b]	.000	1	1.000		
Likelihood Ratio	.102	1	.750		
Fisher's Exact Test				1.000	.615
Linear-by-Linear Association	.095	1	.758		
N of Valid Cases	51				

a. 2 cells (50.0%) have expected count less than 5. The minimum expected count is 1.29.

b. Computed only for a 2x2 table

Reproductive System Disorders * PV or LP on MGPT

Crosstab

			PV or LP on MGPT		Total
			Yes	No	
Reproductive System Disorders	Yes	Count	0	7	7
		% within PV or LP on MGPT	0.0%	15.6%	13.7%
	No	Count	6	38	44
		% within PV or LP on MGPT	100.0%	84.4%	86.3%
Total		Count	6	45	51
		% within PV or LP on MGPT	100.0%	100.0%	100.0%

Chi-Square Tests

	Value	df	Asymptotic Significance (2-sided)	Exact Sig. (2-sided)	Exact Sig. (1-sided)
Pearson Chi-Square	1.082[a]	1	.298		
Continuity Correction[b]	.167	1	.683		
Likelihood Ratio	1.894	1	.169		
Fisher's Exact Test				.578	.392
Linear-by-Linear Association	1.061	1	.303		
N of Valid Cases	51				

a. 1 cells (25.0%) have expected count less than 5. The minimum expected count is .82.

b. Computed only for a 2x2 table

Osteoarticular / Rheumatologic Diseases * PV or LP on MGPT

Crosstab

			PV or LP on MGPT		Total
			Yes	No	
Osteoarticular / Rheumatologic Diseases	Yes	Count	0	10	10
		% within PV or LP on MGPT	0.0%	22.2%	19.6%
	No	Count	6	35	41
		% within PV or LP on MGPT	100.0%	77.8%	80.4%
Total		Count	6	45	51
		% within PV or LP on MGPT	100.0%	100.0%	100.0%

Chi-Square Tests

	Value	df	Asymptotic Significance (2-sided)	Exact Sig. (2-sided)	Exact Sig. (1-sided)
Pearson Chi-Square	1.659[a]	1	.198		
Continuity Correction[b]	.548	1	.459		
Likelihood Ratio	2.808	1	.094		
Fisher's Exact Test				.331	.250
Linear-by-Linear Association	1.626	1	.202		
N of Valid Cases	51				

a. 2 cells (50.0%) have expected count less than 5. The minimum expected count is 1.18.

b. Computed only for a 2x2 table

Alcohol or Tobacco Dependence * PV or LP on MGPT

Crosstab

			PV or LP on MGPT		
			Yes	No	Total
Alcohol or Tobacco Dependence	Yes	Count	0	3	3
		% within PV or LP on MGPT	0.0%	6.7%	5.9%
	No	Count	6	42	48
		% within PV or LP on MGPT	100.0%	93.3%	94.1%
Total		Count	6	45	51
		% within PV or LP on MGPT	100.0%	100.0%	100.0%

Chi-Square Tests

	Value	df	Asymptotic Significance (2-sided)	Exact Sig. (2-sided)	Exact Sig. (1-sided)
Pearson Chi-Square	.425[a]	1	.514		
Continuity Correction[b]	.000	1	1.000		
Likelihood Ratio	.776	1	.379		
Fisher's Exact Test				1.000	.681
Linear-by-Linear Association	.417	1	.519		
N of Valid Cases	51				

a. 2 cells (50.0%) have expected count less than 5. The minimum expected count is .35.

b. Computed only for a 2x2 table
