# Supplementary material for: Multigene Germline Panel Testing in Gastric Cancer Patients in a Portuguese Population
Source: Cancer Med. 2026 Mar 19;15(3):e71732. doi: 10.1002/cam4.71732 (PMC13093424; doi:10.1002/cam4.71732)
Supplement: Supplementary file 10 — Data S10: Supporting Information. [file CAM4-15-e71732-s013.pdf]

other cancers in the same patient * PV or LP on MGPT Crosstabulation

			PV or LP on MGPT		
			Yes	No	Total
other cancers in the same patient	Yes	Count	3	8	11
		% within PV or LP on MGPT	50.0%	17.8%	21.6%
	No	Count	3	37	40
		% within PV or LP on MGPT	50.0%	82.2%	78.4%
Total		Count	6	45	51
		% within PV or LP on MGPT	100.0%	100.0%	100.0%

Chi-Square Tests

	Value	df	Asymptotic Significance (2-sided)	Exact Sig. (2-sided)	Exact Sig. (1-sided)
Pearson Chi-Square	3.249[a]	1	.071		
Continuity Correction[b]	1.624	1	.203		
Likelihood Ratio	2.744	1	.098		
Fisher's Exact Test				.106	.106
Linear-by-Linear Association	3.186	1	.074		
N of Valid Cases	51				

a. 2 cells (50.0%) have expected count less than 5. The minimum expected count is 1.29.

b. Computed only for a 2x2 table
