# Supplementary material for: Multigene Germline Panel Testing in Gastric Cancer Patients in a Portuguese Population
Source: Cancer Med. 2026 Mar 19;15(3):e71732. doi: 10.1002/cam4.71732 (PMC13093424; doi:10.1002/cam4.71732)
Supplement: Supplementary file 11 — Data S11: Supporting Information. [file CAM4-15-e71732-s008.pdf]

Case Processing Summary

	Cases					
	Valid		Missing		Total	
	N	Percent	N	Percent	N	Percent
Direct relatives diagnosed with cancer * PV or LP on MGPT	51	100.0%	0	0.0%	51	100.0%
More_than_1_relative * PV or LP on MGPT	51	100.0%	0	0.0%	51	100.0%
Famlial_Stomach_cancer * PV or LP on MGPT	51	100.0%	0	0.0%	51	100.0%

Direct relatives diagnosed with cancer * PV or LP on MGPT

Crosstab

			PV or LP on MGPT		Total
			Yes	No	
Direct relatives diagnosed with cancer	Yes	Count	4	27	31
		% within PV or LP on MGPT	66.7%	60.0%	60.8%
	No	Count	2	18	20
		% within PV or LP on MGPT	33.3%	40.0%	39.2%
Total		Count	6	45	51
		% within PV or LP on MGPT	100.0%	100.0%	100.0%

Chi-Square Tests

	Value	df	Asymptotic Significance (2-sided)	Exact Sig. (2-sided)	Exact Sig. (1-sided)
Pearson Chi-Square	.099[a]	1	.753		
Continuity Correction[b]	.000	1	1.000		
Likelihood Ratio	.100	1	.751		
Fisher's Exact Test				1.000	.562
Linear-by-Linear Association	.097	1	.756		
N of Valid Cases	51				

a. 2 cells (50.0%) have expected count less than 5. The minimum expected count is 2.35.

b. Computed only for a 2x2 table

More_than_1_relative * PV or LP on MGPT

Crosstab

			PV or LP on MGPT		Total
			Yes	No	
More_than_1_relative	Yes	Count	1	17	18
		% within PV or LP on MGPT	16.7%	37.8%	35.3%
	No	Count	5	28	33
		% within PV or LP on MGPT	83.3%	62.2%	64.7%
Total		Count	6	45	51
		% within PV or LP on MGPT	100.0%	100.0%	100.0%

Chi-Square Tests

	Value	df	Asymptotic Significance (2-sided)	Exact Sig. (2-sided)	Exact Sig. (1-sided)
Pearson Chi-Square	1.033[a]	1	.309		
Continuity Correction[b]	.316	1	.574		
Likelihood Ratio	1.150	1	.284		
Fisher's Exact Test				.405	.299
Linear-by-Linear Association	1.013	1	.314		
N of Valid Cases	51				

a. 2 cells (50.0%) have expected count less than 5. The minimum expected count is 2.12.

b. Computed only for a 2x2 table

Famlial_Stomach_cancer * PV or LP on MGPT

Crosstab

			PV or LP on MGPT		Total
			Yes	No	
Famlial_Stomach_cancer	Yes	Count	1	10	11
		% within PV or LP on MGPT	16.7%	22.2%	21.6%
	No	Count	5	35	40
		% within PV or LP on MGPT	83.3%	77.8%	78.4%
Total		Count	6	45	51
		% within PV or LP on MGPT	100.0%	100.0%	100.0%

Chi-Square Tests

	Value	df	Asymptotic Significance (2-sided)	Exact Sig. (2-sided)	Exact Sig. (1-sided)
Pearson Chi-Square	.097[a]	1	.756		
Continuity Correction[b]	.000	1	1.000		
Likelihood Ratio	.102	1	.750		
Fisher's Exact Test				1.000	.615
Linear-by-Linear Association	.095	1	.758		
N of Valid Cases	51				

a. 2 cells (50.0%) have expected count less than 5. The minimum expected count is 1.29.

b. Computed only for a 2x2 table
