# Supplementary material for: Multigene Germline Panel Testing in Gastric Cancer Patients in a Portuguese Population
Source: Cancer Med. 2026 Mar 19;15(3):e71732. doi: 10.1002/cam4.71732 (PMC13093424; doi:10.1002/cam4.71732)
Supplement: Supplementary file 12 — Data S12: Supporting Information. [file CAM4-15-e71732-s004.pdf]

Histology_resume * PV or LP on MGPT Crosstabulation

			PV or LP on MGPT		
			Yes	No	Total
Histology_resume	Diffuse	Count	1	9	10
		% within PV or LP on MGPT	16.7%	20.0%	19.6%
	Intestinal	Count	3	34	37
		% within PV or LP on MGPT	50.0%	75.6%	72.5%
	Mixed	Count	2	2	4
		% within PV or LP on MGPT	33.3%	4.4%	7.8%
Total		Count	6	45	51
		% within PV or LP on MGPT	100.0%	100.0%	100.0%

Chi-Square Tests

	Value	df	Asymptotic Significance (2-sided)
Pearson Chi-Square	6.140[a]	2	.046
Likelihood Ratio	4.075	2	.130
N of Valid Cases	51		

a. 4 cells (66.7%) have expected count less than 5. The minimum expected count is .47.

Crosstabs
