# Supplementary material for: Multigene Germline Panel Testing in Gastric Cancer Patients in a Portuguese Population
Source: Cancer Med. 2026 Mar 19;15(3):e71732. doi: 10.1002/cam4.71732 (PMC13093424; doi:10.1002/cam4.71732)
Supplement: Supplementary file 13 — Data S13: Supporting Information. [file CAM4-15-e71732-s018.pdf]

Tumour located in antrum vs other locations * PV or LP on MGPT Crosstabulation

			PV or LP on MGPT		
			Yes	No	Total
Tumour located in antrum vs other locations		Count	1	0	1
		% within PV or LP on MGPT	16.7%	0.0%	2.0%
	1	Count	3	21	24
		% within PV or LP on MGPT	50.0%	46.7%	47.1%
	2	Count	2	24	26
		% within PV or LP on MGPT	33.3%	53.3%	51.0%
Total		Count	6	45	51
		% within PV or LP on MGPT	100.0%	100.0%	100.0%

Chi-Square Tests

	Value	df	Asymptotic Significance (2-sided)
Pearson Chi-Square	7.928[a]	2	.019
Likelihood Ratio	4.759	2	.093
N of Valid Cases	51		

a. 4 cells (66.7%) have expected count less than 5. The minimum expected count is .12.
