# Supplementary material for: Multigene Germline Panel Testing in Gastric Cancer Patients in a Portuguese Population
Source: Cancer Med. 2026 Mar 19;15(3):e71732. doi: 10.1002/cam4.71732 (PMC13093424; doi:10.1002/cam4.71732)
Supplement: Supplementary file 14 — Data S14: Supporting Information. [file CAM4-15-e71732-s019.pdf]

cStage_resume * PV or LP on MGPT Crosstabulation

			PV or LP on MGPT		Total
			Yes	No	
cStage_resume	.	Count	1	1	2
		% within PV or LP on MGPT	16.7%	2.2%	3.9%
	Stage I-III	Count	3	37	40
		% within PV or LP on MGPT	50.0%	82.2%	78.4%
	Stage IV	Count	2	7	9
		% within PV or LP on MGPT	33.3%	15.6%	17.6%
Total		Count	6	45	51
		% within PV or LP on MGPT	100.0%	100.0%	100.0%

Chi-Square Tests

	Value	df	Asymptotic Significance (2-sided)	Exact Sig. (2-sided)
Pearson Chi-Square	4.466[a]	2	.107	.111
Likelihood Ratio	3.327	2	.189	.184
Fisher-Freeman-Halton Exact Test	4.670			.065
N of Valid Cases	51			

a. 4 cells (66.7%) have expected count less than 5. The minimum expected count is .24.
