# Supplementary material for: Multigene Germline Panel Testing in Gastric Cancer Patients in a Portuguese Population
Source: Cancer Med. 2026 Mar 19;15(3):e71732. doi: 10.1002/cam4.71732 (PMC13093424; doi:10.1002/cam4.71732)
Supplement: Supplementary file 15 — Data S15: Supporting Information. [file CAM4-15-e71732-s021.pdf]

Subtotal gastrectomy * PV or LP on MGPT Crosstabulation

			PV or LP on MGPT	
			Yes	No
Subtotal gastrectomy	No surgery	Count	1	9
		% within PV or LP on MGPT	16.7%	20.0%
	Subtotal gastrectomy	Count	4	32
		% within PV or LP on MGPT	66.7%	71.1%
	Total gastrectomy	Count	1	4
		% within PV or LP on MGPT	16.7%	8.9%
Total		Count	6	45
		% within PV or LP on MGPT	100.0%	100.0%

Subtotal gastrectomy * PV or LP on MGPT Crosstabulation

			Total
Subtotal gastrectomy	No surgery	Count	10
		% within PV or LP on MGPT	19.6%
	Subtotal gastrectomy	Count	36
		% within PV or LP on MGPT	70.6%
	Total gastrectomy	Count	5
		% within PV or LP on MGPT	9.8%
Total		Count	51
		% within PV or LP on MGPT	100.0%

Chi-Square Tests

	Value	df	Asymptotic Significance (2-sided)	Exact Sig. (2-sided)	Exact Sig. (1-sided)
Pearson Chi-Square	.371[a]	2	.830	1.000	
Likelihood Ratio	.324	2	.850	1.000	
Fisher-Freeman-Halton Exact Test	.912			.791	
Linear-by-Linear Association	.225[b]	1	.635	.699	.473
N of Valid Cases	51				

Chi-Square Tests

	Point Probability
Pearson Chi-Square	
Likelihood Ratio	
Fisher-Freeman-Halton Exact Test	
Linear-by-Linear Association	.288
N of Valid Cases	

a. 4 cells (66.7%) have expected count less than 5. The minimum expected count is .59.

b. The standardized statistic is -.475.
