# Supplementary material for: Multigene Germline Panel Testing in Gastric Cancer Patients in a Portuguese Population
Source: Cancer Med. 2026 Mar 19;15(3):e71732. doi: 10.1002/cam4.71732 (PMC13093424; doi:10.1002/cam4.71732)
Supplement: Supplementary file 16 — Data S16: Supporting Information. [file CAM4-15-e71732-s011.pdf]

QT * PV or LP on MGPT Crosstabulation

			PV or LP on MGPT		
			Yes	No	Total
QT	Yes	Count	4	32	36
		% within PV or LP on MGPT	66.7%	71.1%	70.6%
	No	Count	2	13	15
		% within PV or LP on MGPT	33.3%	28.9%	29.4%
Total		Count	6	45	51
		% within PV or LP on MGPT	100.0%	100.0%	100.0%

Chi-Square Tests

	Value	df	Asymptotic Significance (2-sided)	Exact Sig. (2-sided)	Exact Sig. (1-sided)
Pearson Chi-Square	.050[a]	1	.822	1.000	.578
Continuity Correction[b]	.000	1	1.000		
Likelihood Ratio	.049	1	.824	1.000	.578
Fisher's Exact Test				1.000	.578
Linear-by-Linear Association	.049[c]	1	.824	1.000	.578
N of Valid Cases	51				

Chi-Square Tests

	Point Probability
Pearson Chi-Square	
Continuity Correction[b]	
Likelihood Ratio	
Fisher's Exact Test	
Linear-by-Linear Association	.343
N of Valid Cases	

a. 2 cells (50.0%) have expected count less than 5. The minimum expected count is 1.76.

b. Computed only for a 2x2 table

c. The standardized statistic is -.222.
