# Supplementary material for: Multigene Germline Panel Testing in Gastric Cancer Patients in a Portuguese Population
Source: Cancer Med. 2026 Mar 19;15(3):e71732. doi: 10.1002/cam4.71732 (PMC13093424; doi:10.1002/cam4.71732)
Supplement: Supplementary file 17 — Data S17: Supporting Information. [file CAM4-15-e71732-s005.pdf]

12-month survival * PV or LP on MGPT Crosstabulation

			PV or LP on MGPT		Total
			Yes	No	
12-month survival	Not Yet	Count	0	8	8
		% within PV or LP on MGPT	0.0%	17.8%	15.7%
	Yes	Count	4	34	38
		% within PV or LP on MGPT	66.7%	75.6%	74.5%
	No	Count	2	3	5
		% within PV or LP on MGPT	33.3%	6.7%	9.8%
Total		Count	6	45	51
		% within PV or LP on MGPT	100.0%	100.0%	100.0%

Chi-Square Tests

	Value	df	Asymptotic Significance (2-sided)
Pearson Chi-Square	4.963[a]	2	.084
Likelihood Ratio	4.642	2	.098
Linear-by-Linear Association	4.077	1	.043
N of Valid Cases	51		

a. 4 cells (66.7%) have expected count less than 5. The minimum expected count is .59.
