# Supplementary material for: Multigene Germline Panel Testing in Gastric Cancer Patients in a Portuguese Population
Source: Cancer Med. 2026 Mar 19;15(3):e71732. doi: 10.1002/cam4.71732 (PMC13093424; doi:10.1002/cam4.71732)
Supplement: Supplementary file 20 — Data S20: Supporting Information. [file CAM4-15-e71732-s012.pdf]

NOVA MEDICAL SCHOOL

## DECISÃO FINAL SOBRE O PROJETO

"Multigene Panel Test in Patients in Gastric Cancer Patients in Portugal"

A Comissão de Ética da NMS|FCM-UNL (CEFCM) decidiu aprovar por unanimidade, do ponto de vista ético, o projeto o projeto de investigação intitulado "Multigene Panel Test in Patients in Gastric Cancer Patients in Portugal" (n.º 006/2025/CEFCM), submetido pela doutoranda, Maria Beatriz Mourato, no âmbito do Doutoramento em Ciências e Tecnologias da Saúde e Bem-Estar, da NOVA Medical School.

Lisboa, 24 de abril de 2025

O Presidente da Comissão de Ética,

(Professor Doutor Diogo Pais)

## TO WHOM IT MAY CONCERN

The Ethics Research Committee of NMS|FCM-UNL (CEFCM) has unanimously approved the request corresponding to the project entitled "Multigene Panel Test in Patients in Gastric Cancer Patients in Portugal" (n.º 006/2025/CEFCM), submitted by PhD student Maria Beatriz Mourato in the scope of the PhD in Health and Wellbeing Sciences and Technologies at NOVA Medical School.

Lisbon, April 24th , 2025

The Chairman of the Ethics Research Committee,

(Diogo Pais, MD, PhD)

Campo Mártires da Pátria, 130
1169-056 Lisboa · Portugal
www.nms.unl.pt
