# Supplementary material for: Multigene Germline Panel Testing in Gastric Cancer Patients in a Portuguese Population
Source: Cancer Med. 2026 Mar 19;15(3):e71732. doi: 10.1002/cam4.71732 (PMC13093424; doi:10.1002/cam4.71732)
Supplement: Supplementary file 21 — Data S21: Supporting Information. [file CAM4-15-e71732-s014.pdf]

16/RC

TC.
Ao CA.
05/12/2024

UNIDADE LOCAL DE SAÚDE
ALTO ALENTEJO

INFORMAÇÃO

N.60/2024, de 04 de dezembro

De:Comissão de Ética

Para: Sr. Vogal Executivo do CA- Dr. Raul Cordeiro

C/C:

Assunto:Estudo: "A importância do Painel Multigénico germline no Cancro Gástrico em Países de Risco Intermédio".

Parecer ___/___/______	Despacho/Deliberação 11 DEZ 2024
	Tomado conhecimento. O CA concorda com a presente informação e aprova a realização do estudo: "A Importância do Painel Multigénico Germlide no Cancro Gástrico em Países de Risco Intermédio".<br><br>Dê-se conhecimento:<br>- À Dr.ª Maria Beatriz Mourato;<br>- À Comissão de Ética.<br><br>ULSAALE, EPE<br>Conselho de Administração<br>Joaquim Araújo, Presidente<br>Vera Escoto, Diretora Clínica-CH<br>Ana Bribsa, Diretora Clínica-CSP<br>Jorge Lourenço Marques, Enfermeiro Diretor<br>Raul Cordeiro, Vogal Executivo<br>Ana Amélia Silva, Vogal Executivo<br><br>Ata 57<br>Doc. 66

O estudo supra referido, foi aprovado pela Comissão de Ética, através da informação n.º 14/2024, de 26 de janeiro e autorizado pelo Conselho de Administração em 07 de fevereiro de 2024.
Contudo, a investigadora Dr.ª Maria Beatriz Oliveira Mourato, submeteu a autorização deste grupo de trabalho uma alteração ao consentimento informado, no sentido da disponibilização dos dados dos participantes para outros estudos de investigação, desde que aprovados pela comissão de ética.
Para o efeito, alteraram o modelo de consentimento informado o qual consta anexo.
Compulsado o mesmo, verifica-se que efetivamente contém toda a informação, para que os participantes possam tomar a sua decisão de forma, livre, consciente e esclarecida.

Conclusões e propostas

Analisadas as alterações que se pretendem efetuar, a CE nada tem a opor, propondo autorização.

A decisão que recair sobre esta informação deverá ser notificada:
- À Dra Maria Beatriz Mourato;
- À Comissão de Ética.

É tudo quanto cumpre informar

P´la Comissão de Ética

Maria Luiza Lopes
Digitally signed by Maria Luiza Lopes
Date: 2024.12.04 16:12:53 Z

Anexo: Requerimento..

CES
1 - A

ULSNA-EPE
SECRETARIADO DA ADMINISTRAÇÃO
ENTRADA Nº 202400991 (nov)
04/12/2024
Conceição Mirante

Pedido de alteração ao estudo

Maria Beatriz Baptista de Oliveira Mourato <Beatriz.Mourato@ulsaale.min-saude.pt>
sáb, 25/05/2024 09:29
Para:ULSNA - Comissão de Ética <comissao.etica@ulsna.min-saude.pt>
Cc:Vera Escoto - Diretora Clínica do C.A. da ULSAALE <veraescoto.ca@ulsaale.min-saude.pt>

1 anexos (386 KB)
MGPT - STROBE PROTOCOL.pdf;

Exmos. Srs.,

Em relação ao estudo **"A importância do Painel Multigénico Germline no Cancro Gástrico em Países de Risco Intermédio"**, previamente aprovado pela Comissão de Ética e pelo CA da ULSAALE, para efeitos de integração do mesmo no meu programa de Doutoramento (Doutoramento em Ciências e Tecnologias da Saúde e Bem-Estar, da Escola Nacional de Saúde Pública / Nova Medical School / Universidade de Évora) e posterior submissão também à Comissão de Ética da Nova Medical School, venho por este meio solicitar a V. Exªs. a revisão do seu protocolo devido às seguintes **alterações major**:

- **Nome do estudo: Multigene Panel Test in Patients in Gastric Cancer Patients in Portugal;**
- **Tipo de estudo: Estudo observacional retrospectivo (deixa de ser caso-controlo);**
- **Língua do protocolo: Inglês;**
- **Protocolo STROBE - detalhado;**
- **Alteração ao Consentimento Informado,** nomeadamente em relação à disponibilização dos dados dos participantes para outros estudos de investigação, desde que aprovados por Comissão de Ética, e ao período mais extenso de conservação dos dados. É também detalhado ao participante o modo de armazenamento dos seus dados e os cuidados com a proteção dos mesmos.

Fico ao dispor para qualquer esclarecimento necessário.
Muito grata pela vossa atenção.

Maria Beatriz Baptista de Oliveira Mourato
Médica Assistente Hospitalar de Cirurgia Geral

UNIDADE LOCAL DE SAÚDE DO NORTE ALENTEJANO-EPE | Hospital Dr. José Maria Grande

SEDE:
Av. de Santo António
7300 - 853 Portalegre
TEL: 245 301 000   FAX: 245 330 359

www.ulsna.min-saude.pt

PENSE ANTES DE IMPRIMIR

CES
1

República Portuguesa

SNS
SERVIÇO NACIONAL DE SAÚDE

[link da Instituição]

ULSNA

Mais Saúde e Qualidade de Vida

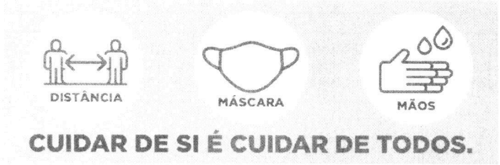

Seja responsável na partilha de informação e/ou dados pessoais nos emails que envia.
Garanta os princípios de confidencialidade, privacidade e proteção de dados.
Lembre-se: os dados salvam vidas, mas o uso abusivo da informação pode destruir a sua vida!
